# Antitumor Activity of Cannabinoids and Their Interaction with Chemotherapy: A Systematic Review and Meta-Analysis of Preclinical Evidence

**DOI:** 10.3390/ph19050768

**Published:** 2026-05-14

**Authors:** Ioana Creangă-Murariu, Ioana-Irina Rezuș, Roshanak Karami, Amir Makolli, Codrin Chifu, Anett Rancz, Zoltán Sipos, Péter Ferdinandy, Renáta Papp, Brigitta Teutsch, Bogdan-Ionel Tamba, Péter Hegyi, Stefania Bunduc

**Affiliations:** 1 Centre for Translational Medicine, Semmelweis University, 1085 Budapest, Hungary; ioana.creanga@d.umfiasi.ro (I.C.-M.);; 2 Advanced Center for Research and Development in Experimental Medicine “Prof. Ostin C. Mungiu”, Grigore T. Popa University of Medicine and Pharmacy, 700115 Iasi, Romania; 3 Medical Oncology-Radiotherapy Department, Grigore T. Popa University of Medicine and Pharmacy, 700115 Iasi, Romania; 4 Department of Radiology, Grigore T. Popa University of Medicine and Pharmacy, 700115 Iasi, Romania; 5 Pharmacy Faculty, Semmelweis University, 1085 Budapest, Hungary; 6 Department of Pharmacology and Pharmacotherapy, Semmelweis University, 1085 Budapest, Hungary; 7 Center for Pharmacology and Drug Research & Development, Semmelweis University, 1085 Budapest, Hungary; 8 Anesthesiology and Intensive Care Unit, Regional Institute of Oncology, 700483 Iasi, Romania; 9 Institute for Translational Medicine, Medical School, University of Pécs, 7622 Pécs, Hungary; 10 Institute of Bioanalysis, Medical School, University of Pécs, 7622 Pécs, Hungary; 11 Pharmahungary Group, 6722 Szeged, Hungary; 12 Department of Radiology, Medical Imaging Centre, Semmelweis University, 1085 Budapest, Hungary; 13 Institute of Pancreatic Diseases, Semmelweis University, 1085 Budapest, Hungary; 14 Translational Pancreatology Research Group, Interdisciplinary Centre of Excellence for Research Development and Innovation, University of Szeged, 6720 Szeged, Hungary; 15 Gastroenterology Department, Carol Davila University of Medicine and Pharmacy, 050474 Bucharest, Romania; 16 Digestive Disease and Liver Transplant Center, Fundeni Clinical Institute, 022328 Bucharest, Romania

**Keywords:** cannabinoids, antitumor effects, glioblastoma, breast cancer, preclinical studies

## Abstract

**Background**: Cannabinoids are studied as anticancer agents, but their effects vary across tumors, compounds, and experimental settings, underscoring the need to define consistent patterns. Our objective was to map cannabinoid efficacy across cancer preclinical models and identify tumor settings with the greatest translational promise. **Methods**: The protocol was registered on PROSPERO (CRD42025543744); PubMed, Embase, and CENTRAL were searched on 4 April 2024 for in vitro and in vivo studies assessing cannabinoid antitumor effects alone or with chemotherapy versus vehicle or chemotherapy only. Random-effects models yielded pooled mean differences (MD) with 95% confidence intervals (CI). MDs of viable cells were calculated for in vitro assays and tumor volume (mm^3^) for in vivo studies. Reports of various compounds, cannabidiol (CBD), tetrahydrocannabinol (THC) or synthetic cannabinoids, were pooled. **Results**: We included 189 studies in the final analysis. In vitro, cannabinoids reduced cell viability modestly overall, with significant effects in glioblastoma (MD −18.77 [CI: −27.15; −10.39]) and a nonsignificant trend in breast cancer (MD −6.75 [CI: −13.90; 0.40]). For in vivo, monotherapy showed the most consistent efficacy in glioblastoma, significantly reducing tumor volume by MD −980.58 mm^3^; [CI: −1270.2; −690.88]. Addition to temozolomide produced a favorable but nonsignificant decrease of MD −220.65 mm^3^; [CI: −579.34; 138.03, vs. temozolomide]. In breast cancer, cannabinoids achieved smaller yet significant tumor reductions (MD −402.64 mm^3^); [CI: −671.84; −133.45]. Synthetic agents had the largest effect (MD −1295.19 mm^3^); [CI: −1664.33; −928.05] -CBD plus doxorubicin vs. doxorubicin). Lung cancer (MD −562.17 mm^3^); [CI: −693.99; −430.35] and prostate cancer (MD −1136.59 mm^3^); [95% CI: −1320.97; −952.21] also had a significant response, whereas colon, pancreatic, and hepatocellular carcinoma models showed inconsistent or null responses. **Conclusions**: Cannabinoids show promise as adjuncts in oncotherapy, particularly in glioblastoma and breast cancer, to enhance chemotherapy efficacy. These findings should be interpreted with caution given the high inter-study heterogeneity typical of preclinical research and should be considered hypothesis-generating, warranting further validation in standardized and clinically relevant models.

## 1. Introduction

Cannabinoids have attracted growing attention in oncology as both supportive agents and potential direct antitumor therapies [1]. Their established role in symptom control, particularly for chemotherapy-induced nausea and vomiting, cancer pain, and anxiety, has led to the clinical approval of synthetic cannabinoids such as dronabinol and nabilone in several countries [2,3]. Reflecting this expanding therapeutic and commercial interest, forecasts estimate that the global cannabis market will reach $82.3 billion by 2027, compared to $27.7 billion in 2022, highlighting a projected annual growth rate of 24.3% [4]. Among Cannabis phytocannabinoids, Δ9-tetrahydrocannabinol (THC) and cannabidiol (CBD) are the most studied and clinically relevant [5]. Despite similar structures, they act through distinct mechanisms and produce contrasting neurobiological effects, shaping both their therapeutic potential and risks [6].

Beyond symptom management, preclinical studies over the past two decades have demonstrated that exogenous cannabinoids can influence key hallmarks of cancer, including proliferation, apoptosis, angiogenesis, and metastasis [7]. Notably, multiple studies indicate that cannabinoids can act synergistically with chemotherapy or radiotherapy, amplifying antitumor effects while potentially attenuating treatment-related toxicity. [8]. These interactions are clinically appealing, as they suggest a capacity to sensitize tumor cells to conventional agents and possibly enable dose reductions that limit systemic adverse effects. Glioblastoma has emerged as the most compelling target, with both consistent preclinical responses and early clinical data indicating improved survival when cannabinoids are combined with temozolomide [9,10].

Despite these promising findings, translation into routine oncology practice remains limited. Human trials investigating cannabinoids for direct antitumor activity are few, small in scale, and heterogeneous in design, with most focusing on palliative rather than disease-modifying outcomes [1]. Moreover, the biological complexity of the endocannabinoid system, variable pharmacology of different cannabinoid compounds, and occasional protumoral effects observed in preclinical models highlight the need for rigorous, indication-specific evidence before broad clinical usage [11].

We aimed to evaluate the antitumor effects of exogenous cannabinoids in preclinical cancer models and to determine their efficacy as monotherapy and in combination with chemotherapy, with the goal of identifying tumor-specific response patterns to inform future clinical translation.

## 2. Results

### 2.1. Characteristics of Included Studies

**Figure 1:**
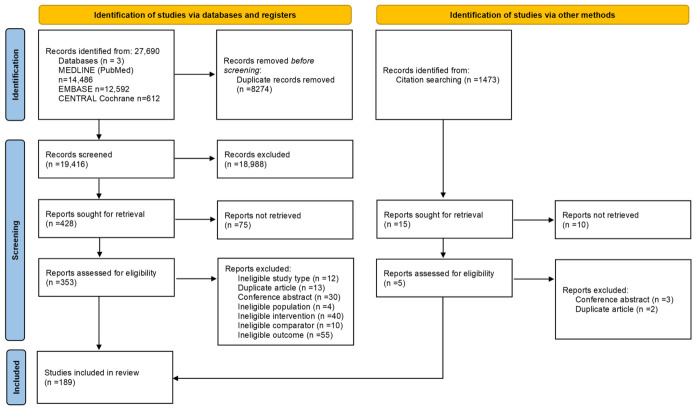
PRISMA flowchart of article selection.

The screening process identified 189 eligible articles from the total of 27,690, of which 52 were included in the meta-analysis (Figure 1). Among these, eight studies reported exclusively in vitro data [12,13,14,15,16,17,18,19], 33 included only animal studies [20,21,22,23,24,25,26,27,28,29,30,31,32,33,34,35,36,37,38,39,40,41,42,43,44,45,46,47,48,49,50,51,52] and 11 combined both approaches [53,54,55,56,57,58,59,60,61,62,63]. Breast cancer emerged as the most frequently investigated tumor type [14,15,16,17,18,19,20,21,22,23,24,25,26,27], followed by glioblastoma [12,13,37,38,39,40,41,42,46,47,48,53]. Digestive tract malignancies were also well represented, with colon cancer examined in six studies [43,50,58,59,60,61], while hepatocarcinoma [35,36,51] and pancreatic cancer were assessed in three studies each. Lung cancer [54,55,56,57] and prostate cancer [31,32,33,34,52] were addressed in four studies each; skin cancers (including melanoma) were explored in three [28,29,30]. Details are presented in Table 1. Phytocannabinoids were more commonly evaluated, either through THC/CBD-rich extracts or preparations with similar cannabinoid ratios [12,13,14,15,16,17,19,20,21,22,23,24,26,27,28,29,33,34,35,36,38,39,40,41,42,43,44,49,51,53,54,55,56,58,60,61,62,63]. Nevertheless, synthetic cannabinoids also featured prominently across several studies [18,19,25,31,32,35,37,41,42,48,51,57,59,63]. Notably, only 16 articles specifically investigated the combined use of cannabinoids with chemotherapy [12,13,14,15,17,18,19,26,27,29,34,40,43,53,54,61]. Characteristics of all eligible studies are listed in Table S3 (in vivo) and Table S4 (in vitro) in Supplementary Materials, together with their key findings, presenting the entire body of evidence on the topic.

### 2.2. Antitumor Effects of Cannabinoids

#### 2.2.1. In Vitro and In Vivo Breast Models

**Figure 2:**
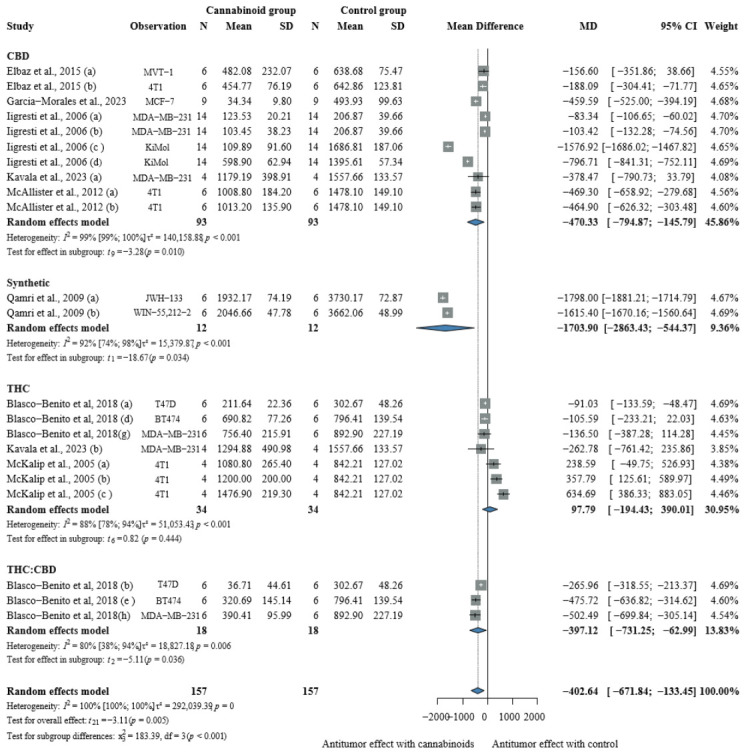
Effects of cannabinoids on breast cancer xenograft tumor volume (mm^3^) [20,21,22,24,25,26,27,64]. Forest plot illustrating the mean difference (MD) in tumor volume between cannabinoid-treated and vehicle-treated animals. Subgroup analyses are stratified by cannabinoid type (CBD, synthetic cannabinoids, THC, and THC:CBD combinations). The “Observation” column provides contextual information on the experimental model or treatment type to aid interpretation of inter-study variability. Abbreviations: CBD = cannabidiol; THC = Δ9-tetrahydrocannabinol; N = sample size; MD = mean difference; SD = standard deviation; CI = confidence interval.

In breast cancer xenograft models, cannabinoid monotherapy demonstrated significant tumor volume reduction compared to vehicle controls, with a pooled MD of −402.64 mm^3^ [CI: −671.84; −133.45], presented in Figure 2.

Subgroup analysis by cannabinoid type revealed that synthetic cannabinoids achieved the best antitumor activity [MD −1703.90 mm^3^; CI: −2863.43; −544.37], while CBD and THC:CBD products exhibited the same trend towards antitumor activity with a −470.33 mm^3^ MD in tumor reduction [CI:−794.87; −145.79] and −397.12 mm^3^ [CI:−731.25; −62.99], respectively. Interestingly, THC-rich products showed a protumoral pattern, with a 97.79 mm^3^ MD in tumor growth [CI:−195.43; 390.01].

The most pronounced tumor control in the case of breast tumor models was achieved when cannabinoids were combined with chemotherapy (Supplementary Figure S3). CBD added to chemotherapy (doxorubicin) produced a marked reduction in tumor volume [−1295.19 mm^3^, CI: −1664.33; −928.05] compared with chemotherapy alone. In contrast, THC monotherapy showed variable results and, on average, performed worse than chemotherapy [MD 263.78 mm^3^, CI: −180.61; 708.16].

Notably, the addition of THC to chemotherapy reversed this pattern, substantially enhancing cytotoxic efficacy [MD −1100.39 mm^3^, CI: −1503.79; −698.99] THC:CBD combinations demonstrated only modest activity as monotherapy [MD 33.77 mm^3^, CI −381.97; 449.51], but yielded clear additional benefit when paired with chemotherapy [MD −70.06 mm^3^, CI: −116.71; −23.40]. When all interventions were pooled, the overall effect was small and statistically nonsignificant [MD −66.39 mm^3^, CI: −343.64; 210.87].

In vitro experiments indicated a modest overall antitumor effect of cannabinoids, though the results varied considerably between studies, reflecting notable heterogeneity (Supplementary Figure S1).

When data were pooled for breast cancer cell lines, cannabinoid exposure was associated with a reduction in cell viability, with MD −6.75 [CI: −13.90; 0.40]. Although this trend suggests a potential inhibitory effect, the result did not reach statistical significance.

#### 2.2.2. In Vitro and In Vivo Glioblastoma Models

**Figure 3:**
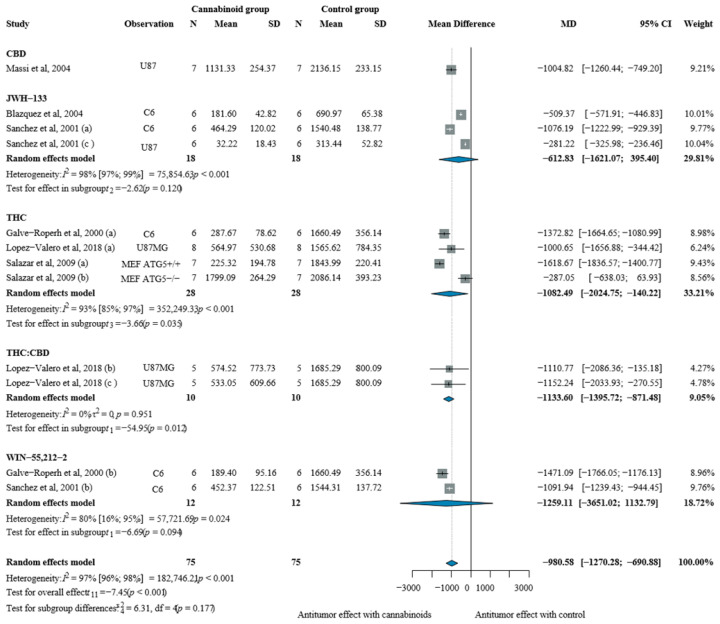
Effects of cannabinoids on glioblastoma xenograft tumor volume (mm^3^) [37,40,41,46,47,48]. Forest plot illustrating the mean difference (MD) in tumor volume between cannabinoid-treated and vehicle-treated animals. Subgroup analyses are stratified by cannabinoid type (CBD, synthetic cannabinoids, THC, and THC:CBD combinations). The “Observation” column provides contextual information on the experimental model to aid interpretation of inter-study variability. Abbreviations: CBD = cannabidiol; THC = Δ9-tetrahydrocannabinol; N = sample size; MD = mean difference; SD = standard deviation; CI = confidence interval.

Glioblastoma monotherapy studies demonstrated significant and consistent tumor growth inhibition across multiple cannabinoid types (Figure 3).

The overall pooled analysis showed substantial tumor volume reduction of −980.58 mm^3^ [CI: −1270.28; −690.88]. Despite high heterogeneity, all cannabinoid categories showed favorable effects.

High THC-based drugs demonstrated significant tumor volume reduction of −1082.49 mm^3^ [CI: −2024.75; −140.22]; the effect was also maintained when drugs contained a combination of THC and CBD [MD −1133.60 mm^3^, CI: −1395.72; −871.48]. Similar antitumor values were also applicable for CBD-rich drugs [−1004.82 mm^3^, CI: −1260.44; −749.20].

Among synthetic cannabinoids, WIN-55,212-2, showed a mean tumor volume reduction of −1259.11 mm^3^ [CI −3651.02; 1132.79] and JWH-133, yielded a pooled effect of −612.83 mm^3^ [CI: −1621.07; 395.40].

Limited combination therapy data were available for glioblastoma models (Supplementary Figure S9). The Lopez-Valero et al. (2018) [40] studies examining cannabinoid–chemotherapy combinations showed tumor volume reduction of −220.65 mm^3^ [CI: −579.34; 138.03] compared to chemotherapy alone, though the effect did not reach statistical significance.

In glioblastoma cell lines, pooled analyses showed a moderate reduction in cell viability with cannabinoids, with MD of −18.77 mm^3^ [CI: −27.15; −10.39]. CBD appeared to exert a more pronounced effect compared to other cannabinoids; however, the overall evidence was marked by very high heterogeneity (Supplementary Figure S7).

#### 2.2.3. In Vivo Lung Cancer Models

**Figure 4:**
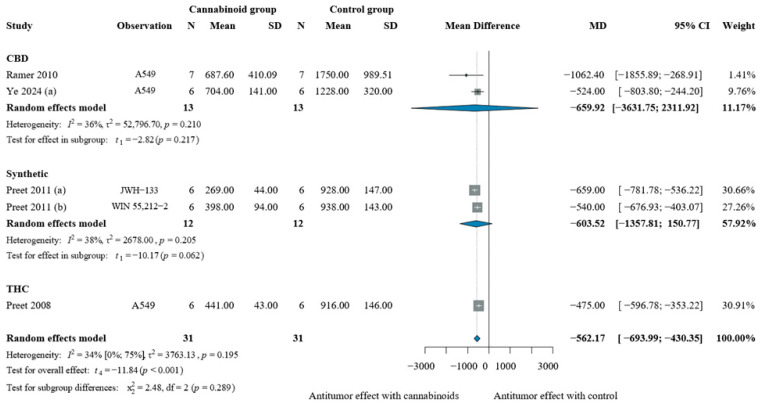
Effects of cannabinoids on lung xenograft tumor volume (mm^3^) [54,55,56,57]. Forest plot illustrating the mean difference (MD) in tumor volume between cannabinoid-treated and vehicle-treated animals. Subgroup analyses are stratified by cannabinoid type (CBD, synthetic cannabinoids, THC, and THC:CBD combinations). The “Observation” column provides contextual information on the experimental model or treatment type to aid interpretation of inter-study variability. Abbreviations: CBD = cannabidiol; THC = Δ9-tetrahydrocannabinol; N = sample size; MD = mean difference; SD = standard deviation; CI = confidence interval.

In lung cancer models, cannabinoids significantly reduced tumor volume compared to vehicle, with a pooled effect of MD −562.17 mm^3^ [CI: −693.99; −430.35], Figure 4. Subgroup analyses showed consistent reductions, with CBD performing best [MD −659.92 mm^3^, CI: −3631.75; 2311.92]; results are seen in Figure 4.

#### 2.2.4. In Vivo Prostate Cancer Models

**Figure 5:**
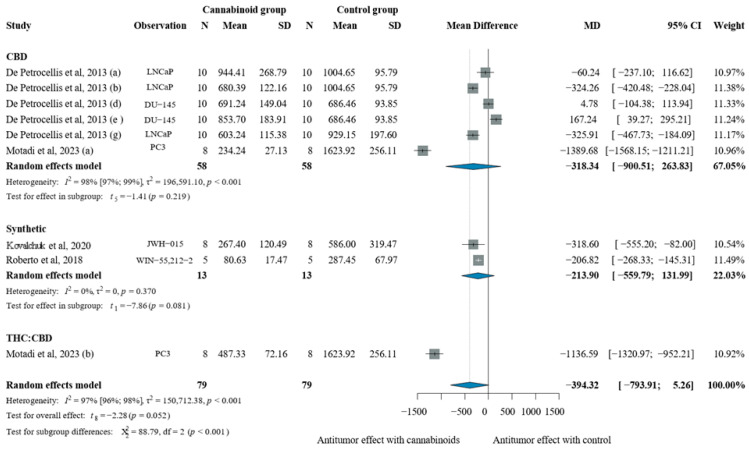
Effects of cannabinoids on prostate xenograft tumor volume (mm^3^) [31,33,34,52]. Forest plot illustrating the mean difference (MD) in tumor volume between cannabinoid-treated and vehicle-treated animals. Subgroup analyses are stratified by cannabinoid type (CBD, synthetic cannabinoids, THC, and THC:CBD combinations). The “Observation” column provides contextual information on the experimental model or treatment type to aid interpretation of inter-study variability. Abbreviations: CBD = cannabidiol; THC = Δ9-tetrahydrocannabinol; N = sample size; MD = mean difference; SD = standard deviation; CI = confidence interval.

When compared to the vehicle, cannabinoids showed a pooled reduction in tumor volume of −394.32 mm^3^ [CI: −793.91; 5.26]. CBD and synthetic cannabinoids demonstrated non-significant reductions, whereas THC:CBD combinations produced a large and significant decrease [MD −1136.59 mm^3^, CI: −1320.97; −952.21], as seen in Figure 5.

Compared with chemotherapy, cannabinoids were generally less effective. CBD alone increased tumor burden [MD 109.78 mm^3^, CI: −185.77; 405.32], while CBD combined with chemotherapy showed a non-significant reduction [MD −157.42 mm^3^, CI: −1121.44; 806.61]. In contrast, THC:CBD with chemotherapy significantly increased tumor growth [MD 306.34 mm^3^, CI: 231.29; 381.39], indicating an antagonistic effect (Supplementary Figure S16).

#### 2.2.5. In Vivo Colon Cancer Models

**Figure 6:**
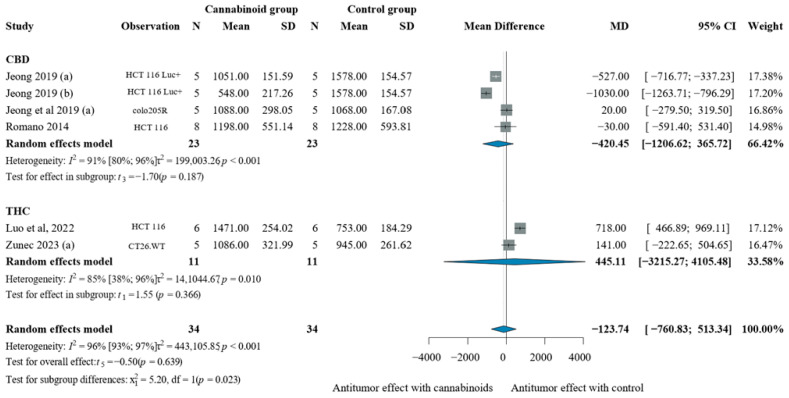
Effects of cannabinoids on colorectal xenograft tumor volume (mm^3^) [43,49,58,60]. Forest plot illustrating the mean difference (MD) in tumor volume between cannabinoid-treated and vehicle-treated animals. Subgroup analyses are stratified by cannabinoid type (CBD, synthetic cannabinoids, THC, and THC:CBD combinations). The “Observation” column provides contextual information on the experimental model to aid interpretation of inter-study variability. Abbreviations: CBD = cannabidiol; THC = Δ9-tetrahydrocannabinol; N = sample size; MD = mean difference; SD = standard deviation; CI = confidence interval.

Colon cancer models showed modest and non-significant responses to cannabinoid monotherapy, with a pooled effect of −123.74 mm^3^ [CI: −760.83; 513.34]. CBD treatment achieved tumor volume reduction of −420.45 mm^3^ [CI: −1206.62; 365.72], though the effect was not statistically significant. THC treatment demonstrated an effect towards tumor growth; however, it was non-significant [MD 445.11 mm^3^, CI: −3215.27; 4105.48], as observed in Figure 6. 

When compared directly to chemotherapy controls, cannabinoids showed worse tumor control in colon cancer models (Supplementary Figure S20). The overall analysis yielded an MD of 130.13 mm^3^ [CI: −398.44; 658.71], indicating larger tumor volumes with cannabinoid treatment compared to chemotherapy. CBD treatment showed minimal effects compared to chemotherapy with an MD of −16.90 mm^3^ [CI: −4011.21; 3977.42], while THC demonstrated poor performance [MD 273.52 mm^3^, CI: −1622.63; 2169.66]. 

### 2.3. No Effect or Protumoral Activity of Cannabinoids

In hepatocellular carcinoma, cannabinoid monotherapy resulted in a minimal but statistically significant absence of effect on tumor volume [MD −2.01 mm^3^; CI: −4.55; 0.53] (Supplementary Figure S24). Pancreatic cancer models showed modest tumor volume reduction, with no statistical significance [MD −50.42 mm^3^, CI: −124.15; 23.32], presented in Supplementary Figure S27.

When combined with chemotherapy, the direction of effect shifted toward tumor activity, though without statistical significance [MD 95.24 mm^3^; CI: −91.81; 282.30] (Supplementary Figure S28). In skin cancer, cannabinoids alone showed a near-neutral pooled effect [MD −2.02 mm^3^; CI: −5.47; 1.43], as shown in Supplementary Figure S32. When combined with other agents, the MD shifted above zero, indicating a nonsignificant trend toward increased tumor volume [MD 47.52 mm^3^; CI: −170.93; 265.98] (Supplementary Figure S33).

### 2.4. Risk of Bias

Across all tumor models evaluated, the overall risk of bias was predominantly high, with a minority of studies classified as some concerns (Supplementary Figures S6, S12, S15, S19, S23, S26, S31 and S36). In vivo experiments were frequently downgraded due to under-reported methodological aspects, especially allocation concealment, blinding, random housing, and outcome selection, which were rarely described beyond routine animal care.

Randomization was often mentioned but lacked details on sequence generation, leading to judgments of some concerns, while baseline comparability at treatment initiation (e.g., tumor volume per group) was seldom documented. Conversely, incomplete outcome data were generally rated as low risk, reflecting the small cohort sizes and limited attrition, and selective reporting was also considered low risk unless discrepancies between planned and reported outcomes were identified.

Similarly, in vitro studies showed consistent downgrades in domains related to sample size calculation, randomization, and blinding, which were typically absent and judged as high risk. Reporting of in vitro model validity parameters, such as cell line authentication, passage number, or mycoplasma testing, was generally limited or absent, which contributed to the overall results. By contrast, outcome measurement validity, statistical analyses, and cell authentication were more robustly reported and usually rated as low risk, as presented in Supplementary Figures S2 and S8. Publication bias presented via funnel plots are seen in Figures S4, S5, S10, S11, S13, S14, S17, S18, S21, S22, S25, S29, S30, S34 and S35.

### 2.5. Heterogeneity

Substantial heterogeneity was observed across all analyses, reflecting variability in tumor types, experimental models, cannabinoid compounds, and treatment regimens. Heterogeneity was quantified using I^2^ and τ^2^ statistics and is reported for each meta-analysis in the corresponding forest plots. Across tumor types, heterogeneity ranged from moderate to high, with values ranging from 34 to 100%, with particularly elevated values observed in analyses involving breast cancer and glioblastoma models. Variability was also evident between in vitro and in vivo studies, as well as across different cannabinoid classes and treatment modalities. Subgroup analyses stratified by tumor type, cannabinoid class, and treatment context (monotherapy versus combination therapy) reduced heterogeneity in some cases, although residual variability remained substantial. Due to the limited number of studies within specific categories and inconsistent reporting of key experimental variables, further stratification was not feasible.

## 3. Discussion

This study provides the most comprehensive systematic synthesis to date of preclinical evidence regarding the antitumor effects of cannabinoids across a wide spectrum of cancer models. A key strength of this work lies in the explicit differentiation between monotherapy and combination strategies, which revealed distinct and clinically relevant activity patterns. Cannabinoids demonstrated consistent and statistically significant antitumor effects in glioblastoma and breast cancer models, with additional supportive evidence in lung and prostate cancer. By contrast, findings were more variable in digestive tract tumors, largely neutral in hepatocellular carcinoma, and occasionally protumoral in colon and some prostate models, particularly when THC was administered in monotherapy.

The therapeutic potential of cannabinoids as adjuvants to chemotherapy emerged most clearly in breast cancer models, where several studies demonstrated that co-administration of CBD or THC enhanced the efficacy of cytotoxic agents. This observation aligns with mechanistic reports indicating that cannabinoids may sensitize tumor cells to chemotherapy through pathways involving autophagy induction, ceramide accumulation, and endoplasmic reticulum stress [65]. Such findings suggest that cannabinoids could function as potentiators rather than replacements of conventional therapy, a role that is clinically attractive given the dose-limiting toxicities associated with standard chemotherapeutics. However, antagonistic interactions were also observed in prostate cancer, highlighting that not all combinations are beneficial and that rigorous preclinical screening is required before clinical translation.

Glioblastoma emerged as the tumor type with the most reproducible evidence base, characterized by consistent monotherapy efficacy and a mechanistic rationale that is coherent across models. This reinforces prior clinical observations: in a phase Ib randomized trial, nabiximols (a THC:CBD extract) in combination with temozolomide improved survival outcomes in patients with recurrent glioblastoma compared with placebo [9]. These converging lines of evidence support glioblastoma as the most compelling candidate for cannabinoid-based therapeutic development. Breast cancer also appears promising, though heterogeneity in outcomes suggests that cannabinoid type, tumor subtype, and receptor expression may modulate efficacy [66]. By contrast, digestive tract cancers, particularly colorectal and pancreatic tumors, demonstrated inconsistent or limited benefit across the studies included in this review, with several reports showing no significant antitumor effect or unfavorable trends following cannabinoid treatment. These discrepancies may reflect differences in receptor distribution, microenvironmental context, or interactions with oncogenic signaling pathways [22].

The divergent activity patterns also highlight the relevance of compound selection. CBD demonstrated the broadest and most favorable profile, with consistent antitumor activity and the advantage of a well-documented safety record in clinical use [2]. Synthetic CB2 agonists also showed promise, though their limited human data necessitates careful evaluation in early-phase studies. In contrast, THC produced highly variable effects, ranging from robust tumor inhibition in glioblastoma to neutral or even protumoral outcomes in breast and colon cancer, consistent with prior reports suggesting that CB1-mediated immunosuppressive signaling may offset direct cytotoxicity [67]. Formulations combining THC and CBD, particularly in ratio-based extracts, may mitigate some of these limitations, though outcomes were again cancer-type dependent.

The strengths of this review lie in its comprehensive synthesis of preclinical evidence on cannabinoid activity in cancer, supported by prospective protocol registration, rigorous methodology, and the explicit distinction between monotherapy and combination strategies. This approach enabled the identification of cancer-specific response patterns and highlighted contexts in which cannabinoids may hold the greatest translational potential. Given the marked variability in experimental designs, tumor models, and cannabinoid formulations, our integrative strategy was intended to capture overarching trends across studies rather than isolate narrowly defined experimental conditions.

However, this breadth comes with important limitations. A major constraint of this synthesis is the substantial heterogeneity observed across included studies, encompassing tumor types, experimental platforms (in vitro vs. in vivo), cannabinoid compounds, dosing regimens, and outcome measures. While this variability reflects the diversity of the preclinical landscape, it complicates the interpretation of pooled estimates. In the absence of robust subgroup analyses, precluded by limited sample sizes and inconsistent reporting, it remains unclear whether the observed antitumor effects represent a broadly applicable pharmacological feature of cannabinoids or are primarily driven by specific experimental contexts.

These findings should be interpreted with caution and considered hypothesis-generating in light of substantial heterogeneity and risk of bias. While effects may be influenced by specific study subsets, consistent directional trends across models support a biologically meaningful, context-dependent antitumor signal.

This limitation is particularly relevant considering the known context-dependent effects of cannabinoids, including biphasic dose–response relationships and tumor-specific signaling interactions. Accordingly, the aggregated findings should not be interpreted as evidence of uniform efficacy across cancer types, but rather as an indicative signal that warrants further investigation through more standardized and stratified experimental designs. Additional limitations relate to the characteristics and quality of the included studies. Most in vivo experiments relied on xenograft models, which restrict the evaluation of immune-mediated mechanisms and limit insights into tumor microenvironment interactions. Furthermore, although combination strategies with chemotherapy appeared promising, they were relatively underrepresented, and mechanistic endpoints were inconsistently reported across tumor types, limiting biological interpretability.

Regarding the risk of bias, studies with high risk were frequently identified, largely driven by incomplete reporting of key domains such as randomization, blinding, and allocation concealment. Given that these assessments often reflected reporting deficiencies rather than confirmed methodological flaws, exclusion-based sensitivity analyses were not deemed informative. Instead, risk of bias was incorporated into the overall interpretation, and the findings should be regarded as hypothesis-generating.

Finally, additional sources of uncertainty should be acknowledged. Small sample sizes and heterogeneous experimental protocols likely contributed to variability in effect sizes, while publication bias cannot be excluded. In vitro findings are further limited by reliance on reported cell line identity, which may be affected by misidentification, genetic drift, or contamination. As reporting of authentication procedures—such as STR profiling, passage number, or mycoplasma testing—was inconsistent, tumor-specific conclusions derived from these models should be interpreted with caution.

Collectively, these limitations reflect broader challenges in preclinical oncology research and underscore the need for improved methodological standardization, transparent reporting, and more reproducible experimental frameworks to enhance translational relevance [68].

From a clinical utility point of view, the implications of these findings are twofold. First, cannabinoids cannot be considered universal anticancer agents, as their effects are context-dependent and occasionally deleterious. Second, where statistically significant and reproducible benefits were observed, most notably in glioblastoma and, to a lesser extent, breast cancer, cannabinoids appear best suited as adjunctive therapies that may enhance the efficacy or tolerability of chemotherapy. This perspective is consistent with emerging clinical evidence: while symptom control trials in advanced cancer have shown mixed results with respect to pain and anxiety [2], antitumor efficacy has only been suggested in small, early-phase studies [9,69]. The translational challenge, therefore, is to bridge the robust mechanistic rationale and promising preclinical findings with carefully designed clinical trials that evaluate not only tumor control but also patient safety and quality of life [70,71].

Future research must prioritize three key areas. First, systematic evaluation of cannabinoid–chemotherapy combinations across multiple tumor types is essential to determine where synergistic effects are most consistent and where antagonism may occur. Second, biomarker-driven approaches, such as profiling CB1/CB2 expression, downstream signaling activity, or metabolic signatures, could guide patient stratification and enhance trial success. Third, rigorous methodological improvements in preclinical studies, including standardized formulations, reproducible dosing regimens, and larger sample sizes with appropriate randomization, are needed to strengthen the translational evidence base.

## 4. Materials and Methods

### 4.1. Protocol, Registration, and Reporting

This systematic review and meta-analysis were conducted in accordance with PRISMA 2020 (Supplementary Table S1) [72] and best practices in preclinical evidence synthesis. The protocol was prospectively registered in PROSPERO (CRD42025543744) [73], and we fully adhered to it. Methods were defined a priori and adhered to the Cochrane Handbook for systematic reviews of interventions [74]. This work was carried out as part of the Systems Education Program at Semmelweis University and conducted within the Translational Medicine (TM) Cycle Framework by the Academia Europaea [70,75].

### 4.2. Review Objectives and Eligibility Criteria

In this review, the population (P) comprised in vitro tumor cell lines of human or animal origin and in vivo tumor-bearing animals, including cell line–derived xenografts, patient-derived xenografts, syngeneic, humanized, orthotopic, genetically engineered, and chemically induced models, without restrictions on species, sex, or tumor site. The intervention (I) included any exogenous cannabinoid, natural or synthetic, irrespective of formulation, dose, schedule, or route of administration, while the comparator (C) consisted of appropriate controls, namely vehicle-treated or untreated groups as negative controls, and conventional chemotherapeutics as positive controls. The outcomes (O) were cell viability in vitro, expressed as percentage survival after cannabinoid exposure, and tumor volume in vivo, measured with calipers or imaging techniques.

We included original experimental studies with separate control groups, whether randomized or non-randomized in design. In vitro studies were eligible if they used tumor cell lines and reported viability, while in vivo studies had to rely on animal tumor models that measured tumor growth or related endpoints. We excluded human studies, spontaneous tumor models, non-cancer models, studies assessing only endogenous cannabinoids, and those without eligible control groups or outcomes. Reviews, conference abstracts, protocols, and studies without accessible full texts were not considered, and in vitro studies focusing exclusively on mechanistic assessments without reporting viability were also excluded.

### 4.3. Information Sources and Search Strategy

On 4 April 2024, we conducted a comprehensive search of MEDLINE (via PubMed), Embase, and Cochrane CENTRAL from their respective inceptions, without imposing language or publication date restrictions. The search strategies integrated terminology related to cannabinoids and cancer, as detailed in the Supplementary Materials (Table S2). The reference lists of all included articles were further checked using citationchaser (Version 2.0, Stockholm Environment Institute, Stockholm, Sweden) [76] on 19 May 2024 to identify eligible articles.

### 4.4. Study Selection

All retrieved records were initially imported into EndNote for duplicate removal and subsequently screened using Rayyan. Screening proceeded in two stages—title/abstract assessment followed by full-text evaluation—conducted independently by three reviewers (IC-M, I-IR, RK). Any disagreements were resolved through discussion or, when necessary, adjudication by a fourth reviewer (AR). Inter-rater reliability was assessed using Cohen’s kappa coefficient.

### 4.5. Data Extraction and Management

Data extraction was performed independently by four independent reviewers (IC-M, I-IR, AM, CC) using a piloted standardized form, with adjudication by a fifth reviewer (AR) in case of disagreement. Graphical data were extracted using the WebPlotDigitizer tool (Version 4.6, Automeris, CA, USA), and study authors were contacted for clarifications or missing data where feasible. Extracted variables included bibliographic details such as author, year, and DOI; experimental model characteristics including animal species, strain, sex, tumor type and site, model classification, and number of animals or replicates; as well as intervention and comparator details including cannabinoid type, receptor affinity, dose, schedule, route, formulation, and co-treatments. For outcomes, we collected mean difference (MD) and standard deviation (SD) values for percentage cell viability in vitro and tumor volumes in vivo.

### 4.6. Risk of Bias Assessment

Risk of bias was independently evaluated by two reviewers (IC-M, I-IR). Animal studies were appraised using the SYRCLE risk-of-bias tool [77], while the risk of bias for in vitro studies was evaluated using a personal approach, adapted from the framework proposed by Vidhi et al., with judgments categorized as low risk, some concerns, or high risk based on the level of reporting and plausibility of implementation, similar to already validated tools [78]. Disagreements were resolved by a third reviewer (AR).

### 4.7. Data Synthesis and Statistical Analysis

Meta-analyses were conducted when at least three studies reported sufficiently comparable data for a given outcome. Continuous outcomes, such as cell viability, tumor volume, and tumor weight, were summarized as mean differences (MD) or standardized mean differences (SMDs) with corresponding 95% confidence intervals (CIs). Because biological and methodological heterogeneity was anticipated, random-effects models were employed for all pooled analyses, while proportions were synthesized using random-intercept logistic regression. Between-study heterogeneity was quantified using τ^2^ and I^2^ statistics [79]. To ensure comparability across experiments, tumor volumes were standardized to cubic millimeters, and cell viability to percentages. Separate analyses were carried out when comparators involved standard chemotherapy rather than a vehicle or no treatment and when interventions consisted of cannabinoid–chemotherapy combinations rather than cannabinoids alone. Subgroup analyses were refined post hoc to explore additional sources of heterogeneity, including differences in the class or type of cannabinoid investigated, and are therefore considered exploratory.

To provide a meaningful estimate, the prediction interval was reported only when the number of available studies was sufficient (e.g., ≥5) and heterogeneity was not excessive. Small-study publication bias was assessed through visual inspection of funnel plots and by applying the appropriate statistical tests: Egger’s test for continuous outcomes, the Pustejovsky test for standardized mean differences, and the Harbord test for dichotomous outcomes [80]. Potential outlier studies were examined using multiple influence diagnostics and graphical approaches, following the recommendations of Harrer et al. [81]. All statistical analyses were calculated by R software v. 4.6 using the meta5 package for basic meta-analysis calculations and plots, and the dmetar6 package for additional influential analysis calculations and plots.

## 5. Conclusions

Cannabinoids show emerging potential as adjuncts in oncological treatment, with relatively consistent signals observed particularly in glioblastoma and breast cancer models. However, given the substantial heterogeneity and predominantly high risk of bias, these findings should be interpreted with caution and considered hypothesis-generating, warranting further rigorous and translationally oriented research.

**Table 1:** Baseline characteristics table.

Tumor Type	Results	Author/Year
Bladder	Unclear	Whynot et al., 2023 [82], Anis et al., 2021 [83]
Breast	Antitumor	Caffarel et al., 2006 [84], Ligresti et al., 2006 [22], McAllister et al., 2012 [23], Murase et al., 2014 [85], Oliveira et al., 2023 [86], Preet et al., 2007 [56], Qamri et al., 2009 [25], Schoeman et al., 2020 [87], Shrivastava et al., 2011 [88], Takeda et al., 2012 [89], Takeda et al., 2013 [90], Tomko et al., 2019 [19], Shrivastava et al., 2011 [88], Mohammadpour et al., 2017 [91]
	Pro-tumor	McKallip et al., 2005 [24]
	Unclear	Caffarel et al., 2006 [84], Hanlon et al., 2016 [92], Ward et al., 2014 [17], Almeida et al., 2023 [14], Amaral et al., 2021 [93], Caffarel et al., 2010 [94], García-Morales et al., 2023 [21], Takeda et al., 2008 [16], Sainz-Cort et al., 2020 [95], von Bueren et al., 2008 [96], Elbaz et al., 2015 [20], Greish et al., 2018 [18], Kalvala et al., 2023 [26], D’Aloia et al., 2022 [15], Surapaneni et al., 2022 [97]
Cervical	Antitumor	Lukhele et al., 2016 [98]
Cholangiocarcinoma	Antitumor	Leelawat et al., 2010 [99], Leelawat et al., 2022 [100], Leelawat et al., 2023 [101], Viereckl et al., 2022 (a) [102], Viereckl et al., 2022 (b) [103]
Chronic myeloid leukemia	Unclear	Maggi et al., 2022 [104]
Colorectal	Antitumor	Alenabi et al., 2021 [105], Beben et al., 2024 [106], Fiore et al., 2018 [107], Gazzerro et al., 2010 [108], Greenhough et al., 2007 [109], Hwang et al., 2023 [110], Lee et al., 2022 [111], Mun et al., 2022 [112], Nallathambi et al., 2018 [113], Pellerito et al., 2014 [114], Raup-Konsavage et al., 2018 [115], Santoro et al., 2009 [116], Feng et al., 2022 [117], Jeong et al., 2019 [50], Jeong et al., 2019 [49]
	Unclear	Thapa et al., 2012 [118], Cerretani et al., 2020 [119], Raup-Konsavage et al., 2020 [120]
Endometrial	Unclear	Fonseca et al., 2018 [121]
	Antitumor	Marinelli et al., 2020 [122], Zhang et al., 2018 [123]
Gastric	Antitumor	Jeong et al., 2019 [61], Ortega et al., 2016 [124], Xian et al., 2010 [125], Xian et al., 2013 [126], Zhang et al., 2019 [127]
	Unclear	Oh et al., 2013 [128], Chen et al., 2021 [129]
Glioblastoma	Antitumor	Cioni et al., 2019 [130], Ellert-Miklaszewska et al., 2021 [131], Esfandiary et al., 2023 [132], Galanti et al., 2008 [133] Kim et al., 2024 [134], Massi et al., 2003 [47], McAllister et al., 2007 [64], Rupprecht et al., 2022 [135], Salazar et al., 2009 [46], Sanchez et al., 2001 [37], Sanchez et al., 1998 [136], Scott et al., 2015 [137], Solinas et al., 2013 [138], Soroceanu et al., 2022 [13], Torres et al., 2011 [53], Wang et al., 2019 [139], Widmer et al., 2008 [140], Nabissi et al., 2015 [141], Deng et al., 2017 [142], Nabissi et al., 2013 [143], Gomez et al., 2002 [144], Goncharov et al., 2005 [145]
	Unclear	Lorente et al., 2011 [38], Marcu et al., 2010 [146], Peeri et al., 2021 [147], Jacobsson et al., 2000 [148]
Head and neck	Antitumor	Blal et al., 2022 [149], Go et al., 2020 [150]
Hepatocarcinoma	Unclear	Giuliano et al., 2008 [151], Hong et al., 2013 [152], Jeon et al., 2023 [153], Rao et al., 2019 [154]
	Antitumor	Shangguan et al., 2021 [36], Vara et al., 2011 [35], Vara et al., 2013 [51]
Leukemia	Unclear	Kampa-Schittenhelm et al., 2016 [155], Powles et al., 2005 [156], Anceschi et al., 2022 [157], Gholizadeh et al., 2019 [158], Olivas-Aguirre et al., 2021 [159], Besser et al., 2023 [160], McKallip et al., 2006 [161], McKallip 2002 [162]
	Antitumor	Scott et al., 2017 [163], Gallotta et al., 2010 [164]
Lung	Antitumor	Ramer et al., 2010 [165], Ramer et al., 2013 [166], Ye et al., 2024 [54], Preet et al., 2011 [57], Park et al., 2022 [167], Vidinsky et al., 2012 [168], Haustein et al., 2014 [169], Li et al., 2024 [170]
	Unclear	Hamad et al., 2021 [171], Grafinger et al., 2019 [172], Sarafian et al., 2002 [173], Sarafian et al., 2003 [174], Hosami et al., 2021 [175], Milian et al., 2020 [176], Müller et al., 2017 [177]
Mantle cell lymphoma	Unclear	Wasik et al., 2011 [178]
Melanoma	Antitumor	Richtig et al., 2023 [29], Simmerman et al., 2019 [28], Mukosi-Motadi et al., 2023 [179], Carpi et al., 2015 [180]
	Unclear	Petrovici et al., 2021 [181]
Mesothelioma	Antitumor	Colvin et al., 2022 [182]
Multiple cancer models	Unclear	Baram et al., 2019 [183], Choi et al., 2008 [184]
Multiple myeloma	Unclear	Morelli et al., 2013 [185]
	Antitumor	Nabissi et al., 2016 [186]
Neuroblastoma	Antitumor	Fisher et al., 2016 [187], Wang et al., 2022 [188], Wojcieszak et al., 2016 [189]
	Unclear	Sánchez-Sánchez et al., 2023 [190], Tomiyama & Funada 2011 [191]
Oral cancer	Antitumor	Loubaki et al., 2022 [192], Semlali et al., 2021 [193]
Osteosarcoma	Unclear	Xu et al., 2022 [194]
	Antitumor	Zhang et al., 2016 [195]
Ovarian carcinoma	Antitumor	Shalev et al., 2022 [196]
	Unclear	Maguire et al., 2021 [197]
Pancreas	Antitumor	Carracedo et al., 2006 [63], Sakarin et al., 2022 [62], Yang et al., 2020 [44], Emhemmed et al., 2022 [198], Fogli et al., 2006 [199], De Petrocellis et al., 2013 [34], Motadi et al., 2023 [33], Olea-Herrero et al., 2009 [32], Roberto et al., 2018 [31]
	Unclear	Garofano et al., 2022 [200], Luongo et al., 2020 [201], Sreevalsan et al., 2011 [202], Mahmoud et al., 2023 [203]
Skin cancer (epidermal SCC)	Antitumor	Llanos Casanova et al., 2003 [30]
Testicular germ cell	Antitumor	Ahmadi et al., 2020 [204]

## Data Availability

The original contributions presented in this study are included in the article/Supplementary Materials. Further inquiries can be directed to the corresponding authors.

## Supplementary Materials

The following supporting information can be downloaded at https://www.mdpi.com/article/10.3390/ph19050768/s1, Figure S1. The effects of cannabinoids vs control in breast cellular models. Figure S2. Risk of bias for in vitro breast cancer studies. Figure S3. Effects of cannabinoids on breast cancer xenograft tumor volume (mm^3^). Figure S4. Funnel plot cannabinoid vs vehicle in breast cancer. Figure S5. Funnel plot cannabinoid vs chemotherapy in breast cancer. Figure S6. Risk of bias for in vivo breast cancer studies. Figure S7. The effects of cannabinoids vs control in glioblastoma cellular models. Figure S8. Risk of bias in vitro for glioblastoma studies. Figure S9. Effects of cannabinoids on glioblastoma xenograft tumor volume (mm^3^). Figure S10. Funnel plot cannabinoid vs vehicle in glioblastoma. Figure S11. Funnel plot cannabinoid vs chemotherapy in glioblastoma. Figure S12. Risk of bias for glioblastoma studies. Figure S13. Funnel plot cannabinoid vs vehicle in lung cancer. Figure S14. Funnel plot cannabinoid vs chemotherapy in lung cancer. Figure S15. Risk of bias for lung cancer studies. Figure S16. Effects of cannabinoids on prostate xenograft tumor volume (mm^3^). Figure S17. Funnel plot cannabinoid vs vehicle in prostate cancer. Figure S18. Funnel plot cannabinoid vs chemotherapy in prostate cancer. Figure S19. Risk of bias for prostate cancer studies. Figure S20. Effects of cannabinoids on colon cancer xenograft tumor volume (mm^3^). Figure S21. Funnel plot cannabinoid vs vehicle in colorectal cancer. Figure S22. Funnel plot cannabinoid vs chemotherapy in colorectal cancer. Figure S23. Risk of bias for colorectal cancer studies. Figure S24. Effects of cannabinoids on hepatocarcinoma xenograft tumor volume (mm^3^). Figure S25. Funnel plot cannabinoid vs vehicle in hepatocarcinoma. Figure S26. Risk of bias for colorectal cancer studies. Figure S27. Effects of cannabinoids on pancreatic xenograft tumor volume (mm^3^). Figure S28. Effects of cannabinoids on pancreatic xenograft tumor volume (mm^3^). Figure S29. Funnel plot cannabinoid vs vehicle in pancreatic cancer. Figure S30. Funnel plot cannabinoid vs chemotherapy in pancreatic cancer. Figure S31. Risk of bias for pancreatic cancer studies. Figure S32. Effects of cannabinoids on skin xenograft tumor volume (mm^3^). Figure S33. Effects of cannabinoids on skin xenograft tumor volume (mm^3^). Figure S34. Funnel plot cannabinoid vs vehicle in skin cancer. Figure S35. Funnel plot cannabinoid vs chemotherapy in skin cancer. Figure S36. Risk of bias for skin cancer studies. Table S1. PRISMA Checklist. Table S2. Search key. Table S3. Characteristics of included in vivo studies. Table S4. Characteristics of included in vitro studies.

## Abbreviations

The following abbreviations are used in this manuscript:

CB1	Cannabinoid Receptor Type 1
CB2	Cannabinoid Receptor Type 2
CBD	Cannabidiol
CENTRAL	Cochrane Central Register of Controlled Trials
CI	Confidence Interval
I^2^	Inconsistency Index
MD	Mean Difference
mm^3^	Cubic millimeters
PRISMA	Preferred Reporting Items for Systematic Reviews and Meta-Analyses
PROSPERO	International Prospective Register of Systematic Reviews
SD	Standard Deviation
SMD	Standardized Mean Difference
SYRCLE	Systematic Review Centre for Laboratory Animal Experimentation
THC	Δ9-tetrahydrocannabinol
TM	Translational Medicine
τ^2^	Between-study Variance
