# Supplementary material for: Antitumor Activity of Cannabinoids and Their Interaction with Chemotherapy: A Systematic Review and Meta-Analysis of Preclinical Evidence
Source: Pharmaceuticals (Basel). 2026 May 14;19(5):768. doi: 10.3390/ph19050768 (PMC13210788; doi:10.3390/ph19050768)
Supplement: Supplementary file 1 [file pharmaceuticals-19-00768-s001.zip › pharmaceuticals-4267071-supplementary.pdf]

Table S1. PRISMA Checklist

Section and Topic	Item #	Checklist item	Location where item is reported
**TITLE**			
Title	1	Identify the report as a systematic review.	1
**ABSTRACT**			
Abstract	2	See the PRISMA 2020 for Abstracts checklist.	1
**INTRODUCTION**			
Rationale	3	Describe the rationale for the review in the context of existing knowledge.	2
Objectives	4	Provide an explicit statement of the objective(s) or question(s) the review addresses.	3
**METHODS**			
Eligibility criteria	5	Specify the inclusion and exclusion criteria for the review and how studies were grouped for the syntheses.	3
Information sources	6	Specify all databases, registers, websites, organisations, reference lists and other sources searched or consulted to identify studies. Specify the date when each source was last searched or consulted.	3
Search strategy	7	Present the full search strategies for all databases, registers and websites, including any filters and limits used.	3
Selection process	8	Specify the methods used to decide whether a study met the inclusion criteria of the review, including how many reviewers screened each record and each report retrieved, whether they worked independently, and if applicable, details of automation tools used in the process.	3
Data collection process	9	Specify the methods used to collect data from reports, including how many reviewers collected data from each report, whether they worked independently, any processes for obtaining or confirming data from study investigators, and if applicable, details of automation tools used in the process.	4
Data items	10a	List and define all outcomes for which data were sought. Specify whether all results that were compatible with each outcome domain in each study were sought (e.g. for all measures, time points, analyses), and if not, the methods used to decide which results to collect.	4
	10b	List and define all other variables for which data were sought (e.g. participant and intervention characteristics, funding sources). Describe any assumptions made about any missing or unclear information.	4
Study risk of bias assessment	11	Specify the methods used to assess risk of bias in the included studies, including details of the tool(s) used, how many reviewers assessed each study and whether they worked independently, and if applicable, details of automation tools used in the process.	4
Effect measures	12	Specify for each outcome the effect measure(s) (e.g. risk ratio, mean difference) used in the synthesis or presentation of results.	4
Synthesis methods	13a	Describe the processes used to decide which studies were eligible for each synthesis (e.g. tabulating the study intervention characteristics and comparing against the planned groups for each synthesis (item #5)).	4
	13b	Describe any methods required to prepare the data for presentation or synthesis, such as handling of missing summary statistics, or data	4

Section and Topic	Item #	Checklist item	Location where item is reported
		conversions.	
	13c	Describe any methods used to tabulate or visually display results of individual studies and syntheses.	4
	13d	Describe any methods used to synthesize results and provide a rationale for the choice(s). If meta-analysis was performed, describe the model(s), method(s) to identify the presence and extent of statistical heterogeneity, and software package(s) used.	4
	13e	Describe any methods used to explore possible causes of heterogeneity among study results (e.g. subgroup analysis, meta-regression).	4
	13f	Describe any sensitivity analyses conducted to assess robustness of the synthesized results.	4
Reporting bias assessment	14	Describe any methods used to assess risk of bias due to missing results in a synthesis (arising from reporting biases).	4
Certainty assessment	15	Describe any methods used to assess certainty (or confidence) in the body of evidence for an outcome.	NA
**RESULTS**			
Study selection	16a	Describe the results of the search and selection process, from the number of records identified in the search to the number of studies included in the review, ideally using a flow diagram.	5
	16b	Cite studies that might appear to meet the inclusion criteria, but which were excluded, and explain why they were excluded.	5
Study characteristics	17	Cite each included study and present its characteristics.	5
Risk of bias in studies	18	Present assessments of risk of bias for each included study.	11
Results of individual studies	19	For all outcomes, present, for each study: (a) summary statistics for each group (where appropriate) and (b) an effect estimate and its precision (e.g. confidence/credible interval), ideally using structured tables or plots.	6-11
Results of syntheses	20a	For each synthesis, briefly summarise the characteristics and risk of bias among contributing studies.	7-11
	20b	Present results of all statistical syntheses conducted. If meta-analysis was done, present for each the summary estimate and its precision (e.g. confidence/credible interval) and measures of statistical heterogeneity. If comparing groups, describe the direction of the effect.	7-11
	20c	Present results of all investigations of possible causes of heterogeneity among study results.	7-11
	20d	Present results of all sensitivity analyses conducted to assess the robustness of the synthesized results.	7-11
Reporting biases	21	Present assessments of risk of bias due to missing results (arising from reporting biases) for each synthesis assessed.	11
Certainty of evidence	22	Present assessments of certainty (or confidence) in the body of evidence for each outcome assessed.	NA

Section and Topic	Item #	Checklist item	Location where item is reported
**DISCUSSION**			
Discussion	23a	Provide a general interpretation of the results in the context of other evidence.	12
	23b	Discuss any limitations of the evidence included in the review.	12
	23c	Discuss any limitations of the review processes used.	13
	23d	Discuss implications of the results for practice, policy, and future research.	13
**OTHER INFORMATION**			
Registration and protocol	24a	Provide registration information for the review, including register name and registration number, or state that the review was not registered.	3
	24b	Indicate where the review protocol can be accessed, or state that a protocol was not prepared.	3
	24c	Describe and explain any amendments to information provided at registration or in the protocol.	3
Support	25	Describe sources of financial or non-financial support for the review, and the role of the funders or sponsors in the review.	15
Competing interests	26	Declare any competing interests of review authors.	15
Availability of data, code and other materials	27	Report which of the following are publicly available and where they can be found: template data collection forms; data extracted from included studies; data used for all analyses; analytic code; any other materials used in the review.	15

Table S2. Search key

Database	Search Key
**PubMed**<br><br>**CENTRAL Cochrane**	(“Cancer” OR Cancer* OR “Neoplasia” OR “Neoplasm” OR Neoplas* OR “Tumor” OR “Tumors” OR “Tumour” OR “Tumours” OR “Malignancy” OR “Malignancies” OR Malignan* OR “Carcinoma” OR Carcin* OR Oncolog* OR “Adenocarcinoma” OR Adenocarcin* OR “Squamous cell carcinoma” OR “Chondrosarcoma” OR Ewing* OR “Histiocytoma” OR “Osteosarcoma” OR “Rhabdomyosarcoma” OR “Leiomyosarcoma” OR “Myxosarcoma” OR “Fibrosarcoma” OR “Fibromyxoid” OR “Myxofibrosarcoma” OR “Liposarcoma” OR “Angiosarcoma” OR “Kaposi” OR Sarcom* OR “Astrocytoma” OR “Glioma” OR “Oligodendroglioma” OR “Ependymoma” OR “Glioblastoma” OR “Medulloblastoma” OR “Neuroblastoma” OR “Blastoma” OR “Retinoblastoma” OR “cholangiocarcinoma” OR “Hepatocarcinoma” OR Hepatocarcinom* OR “Hepatocellular carcinoma” OR “Hepatocellular” carcinom* OR “Carcinoid” OR “Gastrointestinal stromal tumor” OR “Germ cell tumor” OR “Gestational trophoblastic tumor” OR “Wilms tumor” OR “nephroblastoma” OR “teratoma” OR “germinoma” OR “dysgerminoma” OR “seminoma” OR “gonadoblastoma” OR “Melanoma” OR Melanom* OR “Mesothelioma” OR Mesotheliom* OR “non small cell lung cancer” OR “small cell lung cancer” OR “Thymoma” OR leukem* OR leukaem* OR “leukemia” OR “leukaemia” OR lymphoma* OR “leucocythaemia” OR “myelodysplastic syndrome” OR “myelodysplastic syndromes” OR “myeloproliferative” OR Hodgkin*) AND (“Cannabinoids” OR “cannabis” OR cannabi* OR “phytocannabinoid” OR “sativa” OR “indica” OR “bhang” OR “ganja” OR “charas” OR “hashish” OR “hemp” OR “marihuana” OR “marijuana” OR “cannabinol” OR “cannabidiol” OR “CBD” OR “cannabigerol” OR “cannabichromene” OR “cannabigerivarin” OR “cannabidivarin” OR “cannabielsoin” OR “cannabichromevarin” OR “tetrahydrocannabinol” OR tetrahydrocannabi* OR “THC” OR "9 THC" OR “9” tetrahydrocannabi* OR "delta 9 THC" OR “delta 9” tetrahydrocannabi* OR “nabilone” OR “cesamet” OR “canemes” OR “cannador” OR “dronabinol” OR “palmidrol” OR “dexanabinol” OR “levonantradol” OR “nabiximols” OR “epidiolex” OR “Marinol” OR “Sativex” OR endocannabinoid*)
**Embase**	((‘Cancer’ OR Cancer* OR ‘Neoplasia’ OR ‘Neoplasm’ OR Neoplas* OR ‘Tumor’ OR ‘Tumors’ OR ‘Tumour’ OR ‘Tumours’ OR ‘Malignancy’ OR ‘Malignancies’ OR Malignan* OR ‘Carcinoma’ OR Carcin* OR Oncolog* OR ‘Adenocarcinoma’ OR Adenocarcin* OR ‘Squamous cell carcinoma’ OR

	‘Chondrosarcoma’ OR Ewing* OR ‘Histiocytoma’ OR ‘Osteosarcoma’ OR ‘Rhabdomyosarcoma’ OR ‘Leiomyosarcoma’ OR ‘Myxosarcoma’ OR ‘Fibrosarcoma’ OR ‘Fibromyxoid’ OR ‘Myxofibrosarcoma’ OR ‘Liposarcoma’ OR ‘Angiosarcoma’ OR ‘Kaposi’ OR Sarcom* OR ‘Astrocytoma’ OR ‘Glioma’ OR ‘Oligodendroglioma’ OR ‘Ependymoma’ OR ‘Glioblastoma’ OR ‘Medulloblastoma’ OR ‘Neuroblastoma’ OR ‘Blastoma’ OR ‘Retinoblastoma’ OR ‘cholangiocarcinoma’ OR ‘Hepatocarcinoma’ OR Hepatocarcinom* OR ‘Hepatocellular carcinoma’ OR (Hepatocellular carcinom*) OR ‘Carcinoid’ OR ‘Gastrointestinal stromal tumor’ OR ‘Germ cell tumor’ OR ‘Gestational trophoblastic tumor’ OR ‘Wilms tumor’ OR ‘nephroblastoma’ OR ‘teratoma’ OR ‘germinoma’ OR ‘dysgerminoma’ OR ‘seminoma’ OR ‘gonadoblastoma’ OR ‘Melanoma’ OR Melanom* OR ‘Mesothelioma’ OR Mesotheliom* OR ‘non small cell lung cancer’ OR ‘small cell lung cancer’ OR ‘Thymoma’ OR leukem* OR leukaem* OR ‘leukemia’ OR ‘leukaemia’ OR lymphoma* OR ‘leucocythaemia’ OR ‘myelodysplastic syndrome’ OR ‘myelodysplastic syndromes’ OR ‘myeloproliferative’ OR Hodgkin*) AND (‘Cannabinoids’ OR ‘cannabis’ OR cannabi* OR ‘phytocannabinoid’ OR ‘sativa’ OR ‘indica’ OR ‘bhang’ OR ‘ganja’ OR ‘charas’ OR ‘hashish’ OR ‘hemp’ OR ‘marihuana’ OR ‘marijuana’ OR ‘cannabinol’ OR ‘cannabidiol’ OR ‘CBD’ OR ‘cannabigerol’ OR ‘cannabichromene’ OR ‘cannabigerivarin’ OR ‘cannabidivarin’ OR ‘cannabielsoin’ OR ‘cannabichromevarin’ OR ‘tetrahydrocannabinol’ OR tetrahydrocannabi* OR ‘THC’ OR ‘9 THC’ OR (9 tetrahydrocannabi*) OR ‘delta 9 THC’ OR (delta 9 tetrahydrocannabi*) OR ‘nabilone’ OR ‘cesamet’ OR ‘canemes’ OR ‘cannador’ OR ‘dronabinol’ OR ‘palmidrol’ OR ‘dexanabinol’ OR ‘levonantradol’ OR ‘nabiximols’ OR ‘epidiolex’ OR ‘marinol’ OR ‘sativex’ OR endocannabinoid*)):ab,kw,ti

Table S3. Characteristics of included *in vivo* studies.

Author/ year	Tumor type	Cannabinoid	Results
Whynot et al. 2023 (81)	Bladder	CBD; THC; CBC; CBV ± Gemcitabine/Cisplatin	Unclear
Anis et al. 2021 (82)	Bladder	CBC ± THC	Unclear
Caffarel et al, 2006 (83)	Breast	THC	Antitumor
Ligresti et al, 2006 (33)	Breast	CBD, CBG, CBC, THC-acid, CBD-acid; Cannabis extracts (CBD-rich, THC-rich)	Antitumor
McAllister et al, 2012 (34)	Breast	CBD	Antitumor
McKallip et al, 2005 (35)	Breast	Δ9-THC	Pro-tumor
Mohammadpour et al, 2017 (84)	Breast	ACEA, AM251	No effect
Murase et al, 2014 (85)	Breast	CBD, O-1663 (resorcinol derivative)	Antitumor
Oliveira et al, 2023 (86)	Breast	CBD±Somatostatin	Antitumor
Preet et al, 2007 (67)	Breast	WIN-55,212-2 (CB1/CB2 agonist), JWH-133 (CB2 agonist)	Antitumor
Qamri et al, 2009 (36)	Breast	JWH-133 (CB2 agonist), WIN-55,212-2 (CB1/CB2 agonist)	Antitumor
Schoeman et al, 2020 (87)	Breast	THC+CBG+CBN+CBD	Antitumor
Shrivastava et al, 2011 (88)	Breast	CBD	Antitumor
Takeda et al, 2012 (89)	Breast	CBDA	Antitumor
Takeda et al, 2013 (90)	Breast	THC	Antitumor
Tomko et al, 2019 (30)	Breast	Abnormal CBD, O-1602	Antitumor
Caffarel et al, 2006 (83)	Breast	Δ9-THC	Unclear
Hanlon et al. 2016 (91)	Breast	JWH-015 (CB2 agonist)	Unclear
Shrivastava et al, 2011 (88)	Breast	Cannabidiol (CBD)	Antitumor
Takeda et al, 2012 (89)	Breast	Cannabidiolic acid (CBDA)	Antitumor
Ward et al. 2014 (28)	Breast	CBD ± Paclitaxel	Unclear
Almeida et al. 2023 (25)	Breast	CBD ± Aromatase inhibitors (Exemestane, Anastrozole, Letrozole)	Unclear
Amaral et al. 2021 (92)	Breast	CBD; THC; AEA	Unclear
Caffarel et al. 2010 (93)	Breast	THC; JWH-133	Unclear
García-Morales et al. 2023 (32)	Breast	CBD	Unclear
Takeda et al. 2008 (27)	Breast	THC	Unclear
Sainz-Cort et al. 2020 (94)	Breast	CBD	Unclear
von Bueren et al, 2008 (95)	Breast	THC	Unclear

Elbaz et al. 2015 (31)	Breast	CBD	Unclear
Greish et al. 2018 (29)	Breast	WIN55,212-2 (free vs SMA-nanoformulated)	Unclear
Kalvala et al. 2023 (37)	Breast	CBD; THCV ± Doxorubicin	Unclear
Mohammadpour et al, 2017 (84)	Breast	ACEA (CB1 agonist), AM251 (CB1 antagonist)	Antitumor
D'Aloia et al. 2022 (96)	Breast	CBD ± Cisplatin	Unclear
Surapaneni et al. 2022 (97)	Breast	CBD ± Doxorubicin	Unclear
Lukhele el al, 2016 (98)	Cervical	CBD	Antitumor
Leelawat et al, 2010 (99)	Cholangiocarcinoma	THC	Antitumor
Leelawat et al, 2022 (100)	Cholangiocarcinoma	THC, CBD	Antitumor
Leelawat et al, 2023 (101)	Cholangiocarcinoma	CBN	Antitumor
Viereckl et al, 2022 (a) (102)	Cholangiocarcinoma	CBD	Antitumor
Viereckl et al, 2022 (b) (103)	Cholangiocarcinoma	CBG	Antitumor
Maggi et al, 2022 (104)	Chronic myeloid leukemia	CBD	Unclear
Alenabi et al, 2021 (105)	Colorectal	GW833972A	Antitumor
Beben et al, 2024 (106)	Colorectal	CBD, CBD-HQ, CBG	Antitumor
Fiore et al, 2018 (107)	Colorectal	Rimonabant (SR141716) ± 5FU	Antitumor
Gazzerro et al, 2010 (108)	Colorectal	Rimonabant (SR141716) ± Oxaliplatin	Antitumor
Greenhough et al, 2007 (109)	Colorectal	Cannabis	Antitumor
Hwang et al, 2023 (110)	Colorectal	THC	Antitumor
Lee et al, 2022 (111)	Colorectal	CBD	Antitumor
Mun et al, 2022 (112)	Colorectal	C.Sativa extract	Antitumor
Nallathambi et al, 2018 (113)	Colorectal	C.Sativa extract	Antitumor
Pellerito et al, 2014 (114)	Colorectal	WIN55,212-2	Antitumor
Raup-Konsavage et al, 2018 (115)	Colorectal	THC, CBD and other synthetic cannabinoids	Antitumor
Santoro et al, 2009 (116)	Colorectal	Rimonabant (SR141716)	Antitumor
Thapa et al, 2012 (117)	Colorectal	Cannabis	Unclear
Feng et al, 2022 (118)	Colorectal	CBD	Antitumor
Jeong et al, 2019 (61)	Colorectal	CBD	Antitumor
Jeong et al, 2019 (60)	Colorectal	CBD (with oxaliplatin)	Antitumor
Cerretani et al. 2020 (119)	Colorectal	CBD; THC; CB83 (synthetic)	Unclear
Raup-Konsavage et al. 2020 (120)	Colorectal, Melanoma, Glioblastoma	Pure CBD vs CBD oil extracts	Unclear

Fonseca et al, 2018 (121)	Endometrial	CBD, THC	Unclear
Marinelli et al, 2020 (122)	Endometrial	CBD ± Doxorubicin/Cisplatin/Paclitaxecl	Antitumor
Zhang et al, 2018 (123)	Endometrial	THC	Antitumor
Jeong et al, 2019 (72)	Gastric	CBD	Antitumor
Oh et al, 2013 (124)	Gastric	WIN 55,212-2	Unclear
Ortega et al, 2016 (125)	Gastric	CP 55,940	Antitumor
Xian et al, 2010 (126)	Gastric	WIN 55,212-2	Antitumor
Xian et al, 2013 (127)	Gastric	WIN 55,212-3 ± 5FU	Antitumor
Zhang et al, 2019 (128)	Gastric	CBD	Antitumor
Chen et al. 2021 (129)	Gastric	CBD	Unclear
Cioni et al, 2019	Glioblastoma	COR167	Antitumor
Ellert-Miklaszewska et al, 2021 (130)	Glioblastoma	WIN 55,212-2, JWH-133	Antitumor
Esfandiary et al, 2023 (131)	Glioblastoma	C.Sativa extract	Antitumor
Galanti et al, 2008 (132)	Glioblastoma	THC	Antitumor
Kim et al, 2024 (133)	Glioblastoma	CBD	Antitumor
Lorente et al, 2011 (49)	Glioblastoma	THC	Unclear
Marcu et al, 2010 (134)	Glioblastoma	CBD, THC	Unclear
Massi et al, 2003 (58)	Glioblastoma	CBD	Antitumor
McAllister et al, 2007 (135)	Glioblastoma	THC	Antitumor
Peeri et al, 2021 (136)	Glioblastoma	THC, CBD	Unclear
Rupprecht et al, 2022 (137)	Glioblastoma	THC:CBD	Antitumor
Salazar et al, 2009 (57)	Glioblastoma	THC	Antitumor
Sanchez et al, 2001 (48)	Glioblastoma	JWH-133, WIN-55,212-2	Antitumor
Sanchez et al, 1998 (138)	Glioblastoma	THC	Antitumor
Scott et al, 2015 (139)	Glioblastoma	CBD	Antitumor
Solinas et al, 2013 (140)	Glioblastoma	CBD	Antitumor
Soroceanu et al, 2022 (24)	Glioblastoma	CBD ± THC	Antitumor
Torres et al, 2011 (64)	Glioblastoma	THC, CBD, Sativex-like extract	Antitumor
Wang et al, 2019 (141)	Glioblastoma	JWH-133	Antitumor
Widmer et al, 2008 (142)	Glioblastoma	THC	Antitumor
Nabissi et al, 2015 (143)	Glioblastoma	Cannabidiol (CBD)	Antitumor

Deng et al, 2017 (144)	Glioblastoma	CBD	Antitumor
Nabissi et al, 2013 (145)	Glioblastoma	CBD	Antitumor
Gomez et al, 2002 (146)	Glioma	THC	Antitumor
Goncharov et al, 2005 (147)	Glioma	THC	Antitumor
Jacobsson et al, 2000 (148)	Glioma	THC ± Tamoxifen	Unclear
Blal et al, 2022 (149)	Head and Neck	Cannabis extract	Antitumor
Go et al, 2020 (150)	Head and Neck	CBD	Antitumor
Giuliano et al, 2008 (151)	Hepatocarcinoma	WIN55,212-2	Unclear
Hong et al, 2013 (152)	Hepatocarcinoma	WIN55,212-2	Unclear
Jeon et al, 2023 (153)	Hepatocarcinoma	CBD + cabozantinib	Unclear
Rao et al, 2019 (154)	Hepatocarcinoma	Cannabis	Unclear
Shangguan et al, 2021 (47)	Hepatocarcinoma	CBD	Antitumor
Vara et al, 2011 (46)	Hepatocarcinoma	THC, JWH-015	Antitumor
Vara et al, 2013 (62)	Hepatocarcinoma	THC, JWH-015	Antitumor
Kampa-Schittenhelm et al, 2016 (155)	Leukemia	Dronabinol (THC)	Unclear
Powles et al, 2005 (156)	Leukemia	THC	Unclear
Scott et al, 2017 (157)	Leukemia	CBD, THC, CBG (alone and pairs)	Antitumor
Anceschi et al, 2022 (158)	Leukemia	CBD-rich hemp extracts	Unclear
Gholizadeh et al, 2019 (159)	Leukemia	WIN55,212-2; AM251	Unclear
Gallotta et al, 2010 (160)	Leukemia	Rimonabant (SR141716)	Antitumor
Olivas-Aguirre et al, 2021 (161)	Leukemia	CBD + Tamoxifen	Unclear
Besser et al, 2023 (162)	Leukemia	CBD-rich extract (Extract 12)	Unclear
McKallip et al, 2006 (163)	Leukemia	Cannabidiol (CBD)	Unclear
McKallip 2002 (164)	Leukemia	THC; HU-210; Anandamide; JWH-015	Unclear
Ramer et al, 2010 (165)	Lung	CBD	Antitumor
Ramer et al, 2013 (166)	Lung	CBD	Antitumor
Ye et al, 2024 (65)	Lung	CBD (with dasatinib)	Antitumor
Preet et al, 2011 (68)	Lung	WIN-55,212-2 (CB1/CB2 agonist), JWH-015 (CB2 agonist), JWH-133	Antitumor
Hamad et al, 2021 (167)	Lung	CBD	Unclear
Grafinger et al, 2019 (168)	Lung	5F-MDMB-PINACA; ADB-CHMINACA; MDMB-CHMICA; 5C-AKB48; NM-2201	Unclear
Sarafian et al, 2002 (169)	Lung	Δ9-THC; marijuana smoke tar	Unclear

Sarafian et al. 2003 (170)	Lung	THC	Unclear Antitumor
Hosami et al, 2021 (171)	Lung	Cannabis sativa extract (CS); Echinacea purpurea extract (EP)	Unclear
Park et al, 2022 (172)	Lung	CBD	Antitumor
Vidinsky et al, 2012 (173)	Lung	JWH-133	Antitumor
Haustein et al, 2014 (174)	Lung	CBD; THC; R(+)-methanandamide	Antitumor
Milian et al, 2020 (175)	Lung	THC; CBD (± combination 1:1)	Unclear
Li et al, 2024 (176)	Lung	CBD ± CIK cells	Antitumor
Müller et al. 2017 (177)	Lung carcinoma, Testicular cancer, Neuroblastoma	WIN55,212-2	Unclear
Wasik et al, 2011 (178)	Mantle cell lymphoma	WIN55,212-2	Unclear
Richtig et al, 2023 (40)	Melanoma	CBD ± THC	Antitumor
Simmerman et al, 2019 (39)	Melanoma	CBD	Antitumor
Mukosi-Motadi et al. 2023 (179)	Melanoma	Cannabis sativa extract	Antitumor
Carpi et al, 2015 (180)	Melanoma	AM251	Antitumor
Petrovici et al. 2021 (181)	Melanoma	CBD-enriched hemp oil	Unclear
Colvin et al. 2022 (182)	Mesothelioma	CBD	Antitumor
Baram et al. 2019 (183)	Multiple cancer models	Cannabis extracts (various phytocannabinoids)	Unclear
Choi et al. 2008 (184)	Multiple cancers (melanoma, lung, breast, renal, colon)	CBD	Unclear
Morelli et al. 2013 (185)	Multiple Myeloma	CBD ± Bortezomib	Unclear
Nabissi et al. 2016 (186)	Multiple Myeloma	CBD + THC ± Carfilzomib (CFZ)	Antitumor
Fisher et al. 2016 (187)	Neuroblastoma	CBD; THC	Antitumor
Wang et al. 2022 (188)	Neuroblastoma	Cannabinol (CBN)	Antitumor
Sánchez-Sánchez et al. 2023 (189)	Neuroblastoma	CBD-rich Cannabis extracts	Unclear
Wojcieszak et al. 2016 (190)	Neuroblastoma	JWH-133 (CB2 agonist)	Antitumor
Tomiyama & Funada 2011 (191)	Neuroblastoma	CP-55,940; CP-47,497; CP-47,497-C8	Unclear
Loubaki et al, 2022 (192)	Oral cancer	Cannabinoid mixture	Antitumor
Semlali et al, 2021 (193)	Oral cancer	THC	Antitumor
Xu et al. 2022 (194)	Osteosarcoma	CBD	Unclear
Zhang et al. 2016 (195)	Osteosarcoma	WIN55,212-2	Antitumor
Shalev et al. 2022 (196)	Ovarian carcinoma	THC, CBC, CBG, CBN (fractions F5, F7)	Antitumor
Maguire et al. 2021 (197)	Ovarian carcinoma	CBD	Unclear
Carracedo et al, 2006 (74)	Pancreas	THC, WIN-55,212-2	Antitumor

Sakarin et al, 2022 (73)	Pancreas	THC:CBD	Antitumor
Yang et al, 2020 (55)	Pancreas	CBD, THC, CBD+THC	Antitumor
Emhemmed et al. 2022 (198)	Pancreas	CBD-rich Cannabis sativa extract	Antitumor
Fogli et al. 2006 (199)	Pancreas	AM251, WIN-55,212-2, ACEA, JWH-015	Antitumor
Garofano et al. 2022 (200)	Pancreas	CBD	Unclear
Luongo et al. 2020 (201)	Pancreas	CBD	Unclear
De Petrocellis et al, 2013 (45)	Prostate	CBD; non-THC cannabinoids; CBD-BDS; other BDS	Antitumor
Motadi et al, 2023 (44)	Prostate	CBD; Cannabis sativa extract; ± cisplatin; siRBBP6 context	Antitumor
Olea-Herrero et al, 2009 (43)	Prostate	JWH-015	Antitumor
Roberto et al, 2018 (42)	Prostate	WIN 55,212-2	Antitumor
Sreevalsan et al. 2011 (202)	Prostate	Cannabinoids	Unclear
Mahmoud et al. 2023 (203)	Prostate	CBD, CBG	Unclear
Llanos Casanova et al, 2003 (41)	Skin cancer (epidermal SCC)	WIN-55,212-2 (CB1/CB2), JWH-133 (CB2)	Antitumor
Ahmadi et al. 2020 (204)	Testicular germ cell	THC	Antitumor

Table S4. Characteristics of included *in vitro* studies.

Author/ year	Tumor type	Cell line name	Cannabinoid	Dosage
Whynot et al. 2023	Bladder cancer	T24; TCCSUP	CBD; THC; CBC; CBV ± Gemcitabine/Cisplatin	10 uM
Caffarel et al, 2006	Breast	MCF-7 ,T-47D, MDA-MB-231, MDA-MB-468, EVSA-T, SkBr3	THC	1-12 umol/L
Hacer et al, 2022 (a)	Breast	MDA-MB-231	L-759633, ACPA, ACEA	1-250 uM
Ligresti et al, 2006	Breast	MDA-MB-231 xenograft (mice), in vitro MCF-7, MDA-MB-231	CBD, CBG, CBC, THC-acid, CBD-acid; Cannabis extracts (CBD-rich, THC-rich)	5–6.5 mg
McAllister et al, 2012	Breast	MDA-MB-231, 4T1 (murine) syngeneic metastasis models	CBD	1–1.5 µM (in vitro), 1 mg
McKallip et al, 2005	Breast	MCF-7, MDA-MB-231, 4T1 (murine), EMT6	Δ9-THC	12.5–50 mg
Mohammadpour et al, 2017	Breast	MDA-MB-231	ACEA, AM251	0.01-0.5 uM
Murase et al, 2014	Breast	MDA-MB-231, 4T1 (murine), orthotopic and i.v. metastasis models	CBD, O-1663 (resorcinol derivative)	0.3–1 mg
Oliveira et al, 2023	Breast	MDA-MB-231, MCF-7	CBD±Somatostatin	5 uM
Preet et al, 2007	Breast	MDA-MB-231, MDA-MB-231-luc, SCID mice (xenografts)	WIN-55,212-2 (CB1/CB2 agonist), JWH-133 (CB2 agonist)	1-5 uM (in vitro)
Qamri et al, 2009	Breast	MDA-MB-231, MDA-MB-468, MDA-MB-231-luc, PyMT transgenic mouse model	JWH-133 (CB2 agonist), WIN-55,212-2 (CB1/CB2 agonist)	5 mg
Schoeman et al, 2020	Breast	MDA-MB-231, MCF-7	THC+CBG+CBN+CBD	16-64 uM
Shrivastava et al, 2011	Breast	MDA-MB-231	CBD	2.5-10 uM
Taked et al, 2012	Breast	MDA-MB-231	CBDA	5-25 uM
Taked et al, 2013	Breast	MCF-7	THC	1-50 uM
Tomko et al, 2019	Breast	MDA-MB-231 (paclitaxel-resistant), MCF-7 (paclitaxel-resistant), Zebrafish xenograft	Abnormal CBD, O-1602	0-10 uM
Caffarel et al, 2006	Breast cancer	MCF-7, T-47D, MDA-MB-231, EVSA-T, MDA-MB-468, SKBr3	Δ9-THC	1-12 uM
Hanlon et al. 2016	Breast cancer	4T1; MCF-7; 4T1-luc xenografts	JWH-015 (CB2 agonist)	6 mg
Shrivastava et al, 2011	Breast cancer	MDA-MB-231	Cannabidiol (CBD)	1-10 uM
Takeda et al, 2012	Breast cancer	MDA-MB-231	Cannabidiolic acid (CBDA)	5-25 uM
Ward et al. 2014	Breast cancer (CIPN model)	4T1; MDA-MB-231; mouse C57BL/6 (neuropathy model)	CBD ± Paclitaxel	CBD 2.5–10 mg
Almeida et al. 2023	Breast cancer (ER+)	MCF-7aro	CBD ± Aromatase inhibitors (Exemestane, Anastrozole, Letrozole)	CBD 1-5 uM
Amaral et al. 2021	Breast cancer (ER+)	MCF-7aro	CBD; THC; AEA	5-10 uM nan
Takeda et al, 2013	Breast cancer (ER+)	MCF-7	Δ9-THC	1-50 uM

Caffarel et al. 2010	Breast cancer (ErbB2+)	MMTV-neu mouse model; human ErbB2+ tumors	THC; JWH-133	THC 5 mg
García-Morales et al. 2023	Breast cancer (IL-1β-induced EMT model, 6D cells from MCF-7)	MCF-7-derived 6D cells; xenografts in nu/nu mice	CBD	3.14 mg
Takeda et al. 2008	Breast cancer (MCF-7)	MCF-7	THC	5-20 uM nan
Sainz-Cort et al. 2020	Breast cancer (MCF-7, MDA-MB-231)	MCF-7; MDA-MB-231	CBD	1-20 uM nan
von Bueren 2008	Breast cancer (MCF7, MCF7-AR1)	MCF7; MCF7-AR1	THC	10^{-13}-10^{-4}
Elbaz et al. 2015	Breast cancer (TNBC)	SUM159; SCP2; 4T1.2; MVT-1	CBD	3-9 uM in vitro
Greish et al. 2018	Breast cancer (TNBC)	4T1; MDA-MB-231; MCF-7	WIN55,212-2 (free vs SMA-nanoformulated)	WIN 10 mg
Kalvala et al. 2023	Breast cancer (TNBC, DOX-resistant)	MDA-MB-231 DOX-resistant xenografts	CBD; THCV ± Doxorubicin	CBD 10 mg
Mohammadpour et al, 2017	Breast cancer stem cells	MDA-MB-231 CSCs (CD44+/CD24-/ESA+)	ACEA (CB1 agonist), AM251 (CB1 antagonist)	ACEA 50-200 nM
Hacer et al, 2022	Breast cancer, Pancreatic cancer	MDA-MB-231, PANC1	CB2 agonist L-759633; CB1 agonists ACPA, ACEA	1-250 (tested)
Raup-Konsavage et al. 2020	CRC, Melanoma, Glioblastoma	SW480, HCT116; 1205Lu, A375M; T98G, U87MG	Pure CBD vs CBD oil extracts	10 uM
Lukhele el al, 2016 (a)	Cervical	HeLa, SiHa	CBD	50-151 µg/ml
Lukhele el al, 2016 (b)	Cervical	HeLa, SiHa	C.Sativa extract	50-150 µg/ml
Lukhele et al, 2016	Cervical cancer	HeLa, ME-180, SiHa	Cannabidiol (CBD) vs Cannabis sativa crude extract	1.5-3.2 ug/ml (CBD IC50)
Hacer et al, 2022	Cholangiocarcinoma	HuCCT1	THC, CBN	10-100 µM
Leelawat et al, 2010	Cholangiocarcinoma	RMCCA1, HuCCA1, patient derived specimens	THC	5-100 µM
Leelawat et al, 2010	Cholangiocarcinoma	HuCCA1, RMCCA1	Δ9-THC	5-100 uM
Leelawat et al, 2022	Cholangiocarcinoma	HuCCT1	THC	10-100 µM
Leelawat et al, 2022	Cholangiocarcinoma	HuCCA-1, KKU-100, KKU-213, KKU-452; xenograft (BALB/c nude mice)	CBD	10-40 uM (in vitro)
Leelawat et al, 2023	Cholangiocarcinoma	HuCCT2	CBN	10-101 µM
Viereckl et al, 2022 (a)	Cholangiocarcinoma	HuCC-T1, Mz-ChA-1, H69	CBD	6.25-200 µM
Viereckl et al, 2022 (b)	Cholangiocarcinoma	HuCC-T1, Mz-ChA-1, H70	CBG	6.25-201 µM

Viereckl et al, 2022	Cholangiocarcinoma	HuCC-T1, Mz-ChA-1; normal H69 cholangiocytes	CBD, CBG	6.25-200 uM
Maggi 2022	Chronic myeloid leukemia (CML)	K562; KU812; MOLM-6; K562-IR	CBD	10-75 (IC50: 20 K562
Alenabi et al, 2021	Colon	HT-29, HFF	GW833972A	5-50 µM
Beben et al, 2024	Colon	SW-620, 3D cell culture (spheroid)	CBD, CBD-HQ, CBG	1.5-12 µM
Fiore et al, 2018	Colon	HCT116, 3D cell culture	Rimonabant (SR141716) ± 5FU	0.16-10 µM
Gazzerro et al, 2010	Colon	DLD-1	Rimonabant (SR141716) ± Oxaliplatin	0.1-10 µM
Greenhough et al, 2007	Colon	SW480, HCT-15, HT29, Caco-2, HCT116, LS174T, SW620		2.5-12.5 µM
Hwang et al, 2023	Colon	SW480, LoVo	THC	0-80 µM
Lee et al, 2022	Colon	HCT-116, SW480, SW620, Caco-2	CBD	1-40 µM
Mun et al, 2022	Colon	CT26, HCT116	C.Sativa extract	0-4 µM
Nallathambi et al, 2018	Colon	HCT 116, HT-29, Caco-2, CCD-18Co	C.Sativa extract	35-1600 µg/ml
Pellerito et al, 2014	Colon	HT29, HCT116, Caco-2	WIN55,212-2	2-10 µM
Raup-Konsavage et al, 2018	Colon	W480, SW620, HT29, DLD-1, HCT115, LS174, RKO;	THC, CBD and other synthetic cannabinoids	0.1-100 µM
Santoro et al, 2009	Colon	DLD-1, CaCo-2, SW620	Rimonabant (SR141716)	0.1-20 µM
Thapa et al, 2012	Colon	ANIMAL ONLY!!		nan nan
Bęben et al, 2024	Colon cancer	SW-620	CBD, CBG, CBD-HQ, CBG-A; CBD+CBG combination	1.5-12 ug/mL (IC50 3.9-8.2 ug/mL)
Gazzerro et al, 2010	Colon cancer	DLD-1	Rimonabant (SR141716, CB1 antagonist/inverse agonist) ± oxaliplatin	0.5-10 uM
Pellerito et al, 2014	Colon cancer	HT29, HCT116, Caco-2	WIN55,212-2	Varied (low-mid uM) uM
Fiore et al, 2018	Colon cancer stem cells (CRC CSCs)	GTG7 CSCs; HCT116, DLD1; normal colon organoids ex vivo	Rimonabant (SR141716, CB1 antagonist/inverse agonist)	10-20 uM
Feng et al, 2022	Colorectal	HCT116, SW620, DLD-1, xenograft mouse model	CBD	3–15 µM (in vitro), 10–15 mg
Jeong et al, 2019a	Colorectal	HCT116, DLD-1, CRC patient-derived cells, xenografts	CBD	Variable (3–10 µM in vitro, 5 mg
Jeong et al, 2019b	Colorectal	DLD-1 R, Colo205 R (oxaliplatin-resistant)	CBD (with oxaliplatin)	4 uM (in vitro), combined with oxaliplatin
Alenabi et al, 2021	Colorectal cancer	HT-29; control HFF fibroblasts	GW833972A (CB2 agonist) ± SR144528 (CB2 inverse agonist)	5-50 uM
Greenhough et al, 2007	Colorectal cancer	SW480, HCT-15, HT29, Caco-2, HCT116, LS174T, SW620	Δ9-THC	2.5-10 uM
Hwang et al, 2023	Colorectal cancer	SW480, LoVo	Δ9-THC	40 uM

Lee et al, 2022	Colorectal cancer	SW620, SW480, HCT116, Caco-2; normal CCD18CO	CBD ± CBDV, CBG, CBL, CBGV	5-40 uM
Santoro et al, 2009	Colorectal cancer	DLD-1, Caco-2, SW620; AOM-induced mouse CRC (in vivo)	Rimonabant (SR141716, CB1 antagonist/inverse agonist)	0.1-20 uM
Mun et al, 2022	Colorectal cancer (metastasis)	CT26 (murine), HCT116; CCD-18Co normal colon	Supercritical extract of Cannabis sativa (SEC)	1-4 (in vitro)
Thapa et al, 2012	Colorectal cancer xenograft	HT-29 xenograft, CAM model	LYR-8 (novel hexahydrocannabinol analog; no CB1/CB2 affinity)	10 mg
Nallathambi et al, 2018	Colorectal cancer; colon polyps	HCT116, HT29, Caco-2; adenomatous polyp cells	C. sativa extracts (fractions F3–CBGA-rich, F7–THCA-rich); combinations	20-80 ug/mL
Cerretani et al. 2020	Colorectal carcinoma (HT-29)	HT-29	CBD; THC; CB83 (synthetic)	IC50: CB83 1 uM
Fonseca et al, 2018 (a)	Endometrial	Ishikawa, Hec50co	CBD	0.01-25 µM
Fonseca et al, 2018 (b)	Endometrial	Ishikawa, Hec50co	THC	0.01-25 µM
Marinelli et al, 2020	Endometrial	Ishikawa, MFE-280, HEC-1A, PCEM002, PCEM004a, PCEM004b (primary cell lines)	CBD ± Doxorubicin/Cisplatin/Paclitaxecl	0.49-15.72 µg/ml
Zhang et al, 2018	Endometrial	Patient derived specimens	THC	0.1-20 µM
Fonseca et al, 2018	Endometrial cancer	Ishikawa (type I), Hec50co (type II)	AEA, 2-AG, CBD, THC	≥5 uM
Marinelli et al, 2020	Endometrial cancer	Ishikawa, MFE-280, HEC-1a, PCEM002 (type I); PCEM004a/b (type I/II mixed)	CBD	2.4-15.7 ug/mL
Zhang et al, 2018	Endometrial cancer	HEC-1B, An3ca; EC patient tissues	Δ9-THC	0.1-20 uM
Jeong et al, 2019	Gastric	AGS, MKN45, MKN74, SNU638, NCI-N87	CBD	0-10 µM
Oh et al, 2013 ANIMAL ONLY	Gastric	AGS	WIN 55,212-2	nan nan
Ortega et al, 2016	Gastric	AGS	CP 55,940	0.5-5 µM
Xian et al, 2010	Gastric	AGS, MKN-1	WIN 55,212-2	1-10 µM
Xian et al, 2013	Gastric	SNU-620-5FU/1000	WIN 55,212-3 ± 5FU	0.1-100 µM
Zhang et al, 2019	Gastric	SGC-7901	CBD	10-50 µg/ml
Chen et al. 2021	Gastric cancer	AGS; SGC-7901	CBD	5-40 uM nan
Jeong et al, 2019	Gastric cancer	AGS, MKN45, SNU638, NCI-N87; HFE-145 normal gastric cells	CBD	0-10 uM
Xian et al, 2010	Gastric cancer	AGS, MKN-1	WIN55,212-2	0.1-10 uM
Zhang et al, 2019	Gastric cancer	SGC-7901	Cannabidiol (CBD)	5-40 ug/mL
Xian et al, 2013	Gastric cancer (5-FU resistant)	SNU-620-5FU/1000	WIN55,212-2	1-5 uM
Oh et al, 2013	Gastric cancer xenograft	AGS (xenograft in nude mice)	WIN55,212-2 (mixed CB1/CB2 agonist)	7 mg
Cioni et al, 2019	Glioblastoma	Patient derived specimens	COR167	10^-9-10^-5 M

Ellert-Miklaszewska et al, 2021	Glioblastoma	T98G, LN18, LN229, U251MG, U87MG, patient derived specimens	WIN 55,212-2, JWH-133	15
Esfandiary et al, 2023	Glioblastoma	U87	C.Sativa extract	40-280 µg/ml
Esfandiary et al, 2023	Glioblastoma	U87-MG	Cannabis sativa ethanolic extract	40-280 ug/mL
Galanti et al, 2008	Glioblastoma	U251-MG, U87	THC	5-50 µM
Kim et al, 2024	Glioblastoma	U87, U373	CBD	10-50 µM
Lorente et al, 2011	Glioblastoma	U87, T98, HG2, primary cultures, xenografts	THC	2.5-5 uM (in vitro)
Marcu et al, 2010 (a)	Glioblastoma	SF126, U251, U87	CBD	0.1-5.4 µM
Marcu et al, 2010 (b)	Glioblastoma	SF126, U251, U87	THC	0.1-5.5 µM
Marcu et al, 2010 (c)	Glioblastoma	SF126, U251, U87	CBD:THC	0.1-5.6 µM
Massi et al, 2003	Glioblastoma	U87, U373; xenografts in nude mice	CBD	5-40 uM (in vitro)
McAllister et al, 2005	Glioblastoma	SF126, U87-MG, U251, SF188, U373-MG	THC	2 µM
Peeri et al, 2021	Glioblastoma	A172, U87	THC, CBD	0.2-0.8 µg/ml
Rupprecht et al, 2022	Glioblastoma	U251MG, U138MG	THC:CBD	2.5 µM
Salazar et al, 2009	Glioblastoma	U87, other glioma lines, xenografts	THC	6 uM (in vitro)
Sanchez et al, 2001	Glioblastoma	Rat C6 glioma, human astrocytoma xenografts	JWH-133 (CB2 agonist), WIN-55,212-2 (CB1/CB2 agonist)	50 ug/day (local intratumoral) ug/day
Saènchez et al, 1998	Glioblastoma	C6.9	THC	0.25-1 µM
Scott et al, 2015	Glioblastoma	T98G, U87MG	CBD	1-10 µM
Solinas et al, 2013	Glioblastoma	U87-MG, T98G	CBD	2.5-20 µM
Soroceanu et al, 2022	Glioblastoma	U87, U251, GBM163 (patient-derived), intracranial xenografts	CBD ± THC	0.7-2.8 uM (in vitro)
Torres et al, 2011	Glioblastoma	U87, T98, HG19 (primary), xenografts	THC, CBD, Sativex-like extract	THC 15 mg
Wang et al, 2019	Glioblastoma	C6	JWH-133	1-10 µM
Widmer et al, 2008	Glioblastoma	U373MG	THC	0.5-10 µg/ml
Nabissi 2015	Glioblastoma (GSCs)	Glioblastoma stem-like cells (GSC #1, #30, #83)	Cannabidiol (CBD)	0.5-50 (IC50 ~15-19) uM
Deng 2017	Glioblastoma (T98G, U251, U87MG; mouse PDGF-GBM)	T98G; U251; U87MG; mouse PDGF-GBM	CBD	0.3-100 (IC50 3-9) uM
Nabissi 2013	Glioblastoma (U87MG, MZC; patient-derived)	U87MG; MZC; primary GBM cells	CBD	Up to 25 (EC50 Ca2+ influx 22.2) uM
Gomez et al, 2002	Glioma	C6.9, C6.4	THC	0.5 µM
Goncharov et al, 2005	Glioma	C6	THC	0.5-2 µM

Jacobsson et al, 2000 (a)	Glioma	C6	THC ± Tamoxifen	2-10 µM
Jacobsson et al, 2000 (b)	Glioma	C6	CBD ± Tamoxifen	2-11 µM
Giuliano 2008	HCC	HepG2	WIN55,212-2	1-10 uM
Hong 2013	HCC	BEL-7402	WIN55,212-2	nan nan
Jeon 2023	HCC	HepG2; Hep3B	CBD + cabozantinib	20 (CBD) uM
Rao 2019	HCC	Hep3B; HepG2	MDA19	30-40 uM
Blal et al, 2022	Head and Neck	Cal27, Scc25, Scc9, Scc4	Cannabis extract	1-10 µg/ml
Go et al, 2020	Head and Neck	FaDu, SNU899, SCC15, Hep2)	CBD	1-10 µM
Rao et al, 2019	Hepatocarcinoma			nan nan
Shangguan et al, 2021	Hepatocellular carcinoma	HepG2, HUH7, HCCLM3, MHCC97H, xenografts	CBD	20-40 uM (in vitro)
Vara et al, 2011	Hepatocellular carcinoma	HepG2, HuH-7; xenografts	THC, JWH-015 (CB2 agonist)	8 uM (in vitro)
Vara et al, 2013	Hepatocellular carcinoma	HepG2, HuH-7; xenografts	THC, JWH-015	8 uM (in vitro)
Kampa-Schittenhelm 2016	Leukemia (ALL, AML)	Jurkat; MOLM13; MOLM14; HL60; MV4-11; Kasumi1; K562; patient blasts	Dronabinol (THC)	Jurkat IC50 ~15
Powles 2005	Leukemia (CEM, HEL-92, HL60, MOLT-4)	CEM; HL60; HEL-92; MOLT-4	THC	0-100 uM
Scott 2017	Leukemia (CEM, HL60)	CEM; HL60	CBD, THC, CBG (alone and pairs)	1-50 (IC50 CBD 7.8
Anceschi 2022	Leukemia (CML, K562)	K562	CBD-rich hemp extracts	20 ug/ml extract
Gholizadeh 2019	Leukemia (CML, K562)	K562	WIN55,212-2; AM251	WIN: 0.1-6.4
Gallotta 2010	Leukemia (Jurkat, U937)	Jurkat; U937	Rimonabant (SR141716)	1-20 uM
Olivas-Aguirre 2021	Leukemia (T-ALL)	Jurkat; CCRF-CEM	CBD + Tamoxifen	CBD 0-100
Besser 2023	Leukemia (T-ALL, NOTCH1-mutated)	MOLT-4; CCRF-CEM; Jurkat; Loucy; HPB-ALL; DND-41	CBD-rich extract (Extract 12)	1-5 ug/ml
McKallip 2006	Leukemia (human, murine)	Jurkat; Molt-4; EL-4	Cannabidiol (CBD)	2.5-10 (in vitro)
McKallip 2002	Leukemia/Lymphoma (murine + human ALL)	EL-4; LSA; P815; Jurkat; Molt-4; Sup-T1; primary ALL	THC; HU-210; Anandamide; JWH-015	In vitro: 1-20
Ramer et al, 2010	Lung	A549, H460, H358; xenograft A549	CBD	0.1-1 uM (in vitro)
Ramer et al, 2013	Lung	A549, H460, patient-derived primary lung cancer cells; xenografts	CBD	3 uM (in vitro)

Ye et al, 2024	Lung	A549; xenograft (BALB/c nude mice)	CBD (with dasatinib)	CBD 15 µM (in vitro), 25 mg
Preet et al, 2011	Lung (NSCLC)	A549, SW-1573; SCID mouse xenografts, metastasis model	WIN-55,212-2 (CB1/CB2 agonist), JWH-015 (CB2 agonist), JWH-133	1-20 uM (in vitro)
Hamad 2021	Lung cancer (NSCLC A549/H1299; SCLC H69)	A549; H1299; H69; lung cancer stem cell spheres	CBD	0-48 uM
Grafinger 2019	Lung carcinoma (A549)	A549; TR146	5F-MDMB-PINACA; ADB-CHMINACA; MDMB-CHMICA; 5C-AKB48; NM-2201	Varied (reference standards, extracts, smoke condensates) nan
Sarafian 2001	Lung carcinoma (A549)	A549	Δ9-THC; marijuana smoke tar	nan nan
Sarafian et al. 2002	Lung carcinoma (A549)	A549	THC	10-50 uM nan
Müller et al. 2017	Lung carcinoma, Testicular cancer, Neuroblastoma	A549; HoTu-10; IMR-5	WIN55,212-2	5-20 uM nan
Colvin et al. 2022 (Mesothelioma)	Malignant pleural mesothelioma (MSTO-211H, H28, H2452)	MSTO-211H; H28; H2452	CBD	IC50 ~12-20 uM nan
Wasik 2011	Mantle cell lymphoma	Primary MCL; Granta519; Rec1; JeKo; JVM2	WIN55,212-2; Anandamide (AEA)	Up to 10 uM
Richtig et al, 2023	Melanoma	A375, A2058, SK-Mel-28, A375R, UACC-62, Colo-800, SBcl2; NSG mouse xenografts	CBD ± THC; cannabinoid medication (clinically used)	In vitro uM
Simmerman et al, 2019	Melanoma	B16F10 (murine); C57BL/6 mouse model	CBD	5 mg
Mukosi-Motadi et al. 2023 (Melanoma)	Melanoma (A375)	A375 human melanoma cells	Cannabis sativa extract (methanol)	25-200 ug/mL nan
Carpi 2015	Melanoma (A375, BRAF V600E)	A375	AM251 (CB1 antagonist/inverse agonist)	0.1-50 (IC50 ~5.5 at 48h
Petrovici et al. 2021	Melanoma (MeWo), HeLa, HepG2, Osteosarcoma (HOS)	MeWo; HeLa; HepG2; HOS; NHDF (control)	CBD-enriched hemp oil (decarboxylated)	5-30 ug/mL (CBD eq.) nan
Benedicto et al. 2022	Melanoma, Colorectal carcinoma	B16-F10 (melanoma); MCA38 (CRC)	URB447 (CB2 agonist/CB1 antagonist)	10-50 uM nan
Morelli et al. 2013 (Int J Cancer)	Multiple Myeloma	RPMI8226; U266 (±TRPV2 transfected); patient PCs	CBD ± Bortezomib	CBD IC50 ~20 uM
Nabissi et al. 2016 (Oncotarget)	Multiple Myeloma	U266; RPMI	CBD + THC ± Carfilzomib (CFZ)	THC IC50 ~30-40 uM
Baram et al. 2019	Multiple cancer models	Melanoma, breast, glioblastoma cells	Cannabis extracts (various phytocannabinoids)	nan nan

Choi et al. 2008	Multiple cancers (melanoma, lung, breast, renal, colon)	B16; A549; MDA-MB-231; Renca; SNU-C4	CBD	5-80 uM nan
Hosami 2021	NSCLC (A549)	A549	Cannabis sativa extract (CS); Echinacea purpurea extract (EP)	nan nan
Park 2022	NSCLC (A549)	A549	CBD	≥20 (growth inhibition) uM
Vidinsky 2012	NSCLC (A549) + HUVEC (angiogenesis)	A549; HUVEC	JWH-133	10^{-8}-10^{-4} M
Haustein 2014	NSCLC (A549, H460)	A549; H460; patient metastatic cells; BEAS-2B (control)	CBD; THC; R(+)-methanandamide	nan nan
Milian 2020	NSCLC (A549, H460, H1792)	A549; H460; H1792	THC; CBD (± combination 1:1)	10-100 uM
Li 2024	NSCLC (multiple genotypes)	Various NSCLC lines; CIK cells	CBD ± CIK cells	nan nan
Fisher et al. 2016 (Curr Oncol)	Neuroblastoma	SK-N-SH; LAN-1; IMR-32; NUB-6	CBD; THC	0-50 ug/mL in vitro
Wang et al. 2022 (Cancers)	Neuroblastoma (IMR-5, SK-N-AS)	IMR-5 (p53 WT); SK-N-AS (p53 mutant)	Cannabinol (CBN)	IC50: 20.2 uM (IMR-5)
Sánchez-Sánchez et al. 2023 (IJMS)	Neuroblastoma (SH-SY5Y)	SH-SY5Y	CBD-rich Cannabis extracts	Extracts 3.5 ug/mL nan
Wojcieszak et al. 2016 (J Mol Neurosci)	Neuroblastoma (SH-SY5Y)	SH-SY5Y	JWH-133 (CB2 agonist)	10-40 uM nan
Tomiyama & Funada 2011 (Toxicol Lett)	Neuroblastoma × glioma hybrid (NG108-15)	NG108-15 cells	CP-55,940; CP-47,497; CP-47,497-C8	1-30 uM nan
Loubaki et al, 2022	Oral cancer	Ca9-22	Cannabinoid mixture	0.1-2 µg/ml
Semlali et al, 2021	Oral cancer	Ca9-22	THC	0.1-20 µg/ml
Xu et al. 2022	Osteosarcoma	MG63, 143B	CBD	5-40 uM nan
Zhang et al. 2016	Osteosarcoma	MG63, U2OS	WIN55,212-2	5-20 uM nan
Shalev et al. 2022	Ovarian carcinoma	HTB75, HTB161	THC, CBC, CBG, CBN (fractions F5, F7)	Up to 20 ug/mL nan
Sooda et al. 2023	Ovarian carcinoma	A2780, A2780/CP70	CBD, CBG	1 nM - 100 uM nan
Maguire et al. 2021 (Med Cannabis Cannabinoids)	Ovarian carcinoma (SKOV-3)	SKOV-3 human ovarian carcinoma	CBD (natural vs synthetic)	10-50 uM nan
Hacer et al, 2022 (b)	Pancreas	PANC1	L-759633, ACPA, ACEA	1-251 uM
Donadelli et al. 2011	Pancreatic adenocarcinoma	Panc1, CFPAC1, T3M4, PaCa3, PaCa44, MiaPaCa2	GW405833 (CB2), ACPA (CB1), SR141716 (CB1)	uM range nan
Carracedo et al, 2006	Pancreatic cancer	MiaPaCa2, Panc1; xenografts (subcutaneous and intrapancreatic)	THC, WIN-55,212-2	2-3 uM (in vitro)

Sakarin et al, 2022	Pancreatic cancer	Capan-2; xenografts (BALB/c nude mice)	THC:CBD (1:6 extract)	1, 5, 10 mg
Yang et al, 2020	Pancreatic cancer	Human PC cells (various), pancreatic stellate cells (PSCs), murine syngeneic PC model	CBD, THC, CBD+THC (1:1 oil)	CBD 1-10 uM
Yang et al. 2020	Pancreatic cancer	Multiple PC lines; PSC cells	CBD, THC	1-10 uM nan
Emhemmed et al. 2022	Pancreatic cancer (3D spheroids)	AsPC-1 (organoids)	CBD-rich Cannabis sativa extract	80 ug/mL extract nan
Fogli et al. 2006	Pancreatic carcinoma	MIA PaCa-2	AM251, WIN-55,212-2, ACEA, JWH-015	uM (IC50 AM251 ≈ 8.6 uM) nan
Garofano et al. 2022	Pancreatic carcinoma	PANC-1	CBD (low dose)	1-20 uM nan
Luongo et al. 2020	Pancreatic ductal adenocarcinoma	PANC-1, MIA PaCa-2	CBD	1.5-100 uM nan
Zeppa et al. 2024	Pancreatic ductal adenocarcinoma	PANC-1, MIA PaCa-2	CBG	Up to 31 ug/mL (IC50 ~15 ug/mL) nan
De Petrocellis et al, 2013	Prostate cancer	LNCaP, 22Rv1 (AR+), DU-145, PC-3 (AR−); LNCaP & DU-145 xenografts	CBD; non-THC cannabinoids; CBD-BDS; other BDS	1-10 uM (in vitro)
Motadi et al, 2023	Prostate cancer	PC3; PC3 xenograft (mouse)	CBD; Cannabis sativa extract; ± cisplatin; siRBBP6 context	Varied in vitro
Olea-Herrero et al, 2009	Prostate cancer	PC-3, DU-145, LNCaP; PC-3 xenografts	R(+)-Methanandamide; JWH-015 (CB2 agonist)	uM range (in vitro)
Roberto et al, 2018	Prostate cancer	PC3, DU145, LNCaP; PC3 xenograft	WIN 55,212-2	1-30 uM (in vitro)
Sreevalsan et al. 2011	Prostate cancer (LNCaP, PC-3)	LNCaP; PC-3	Cannabinoids (WIN, CP, anandamide)	5-20 uM nan
Mahmoud et al. 2023	Prostate cancer (hormone-refractory)	TRAMP-C2, enzalutamide-resistant HRPC	CBD, CBG	Up to 30 uM nan
Llanos Casanova et al, 2003	Skin cancer (epidermal SCC)	PDV.C57, HaCa4; nude mouse tumors	WIN-55,212-2 (CB1/CB2), JWH-133 (CB2)	Local administration (ug range, not always specified) ug (local)
Ahmadi et al. 2020	Testicular germ cell (mouse)	Mouse testis tissue in vivo	THC	10 mg
D'Aloia et al. 2022	Triple-negative breast cancer (MDA-MB-231)	MDA-MB-231	CBD ± Cisplatin	5-50 uM nan
Surapaneni et al. 2022	Triple-negative breast cancer (MDA-MB-231, MDA-MB-468)	MDA-MB-231; MDA-MB-468; 3D spheroids	CBD ± Doxorubicin	CBD IC50 3.2 uM (2D), 20-34 uM (3D) nan
Anis et al. 2021	Urothelial carcinoma (T24, TCCSUP)	T24; TCCSUP	CBC ± THC	≤10 uM nan

1. Breast cancer

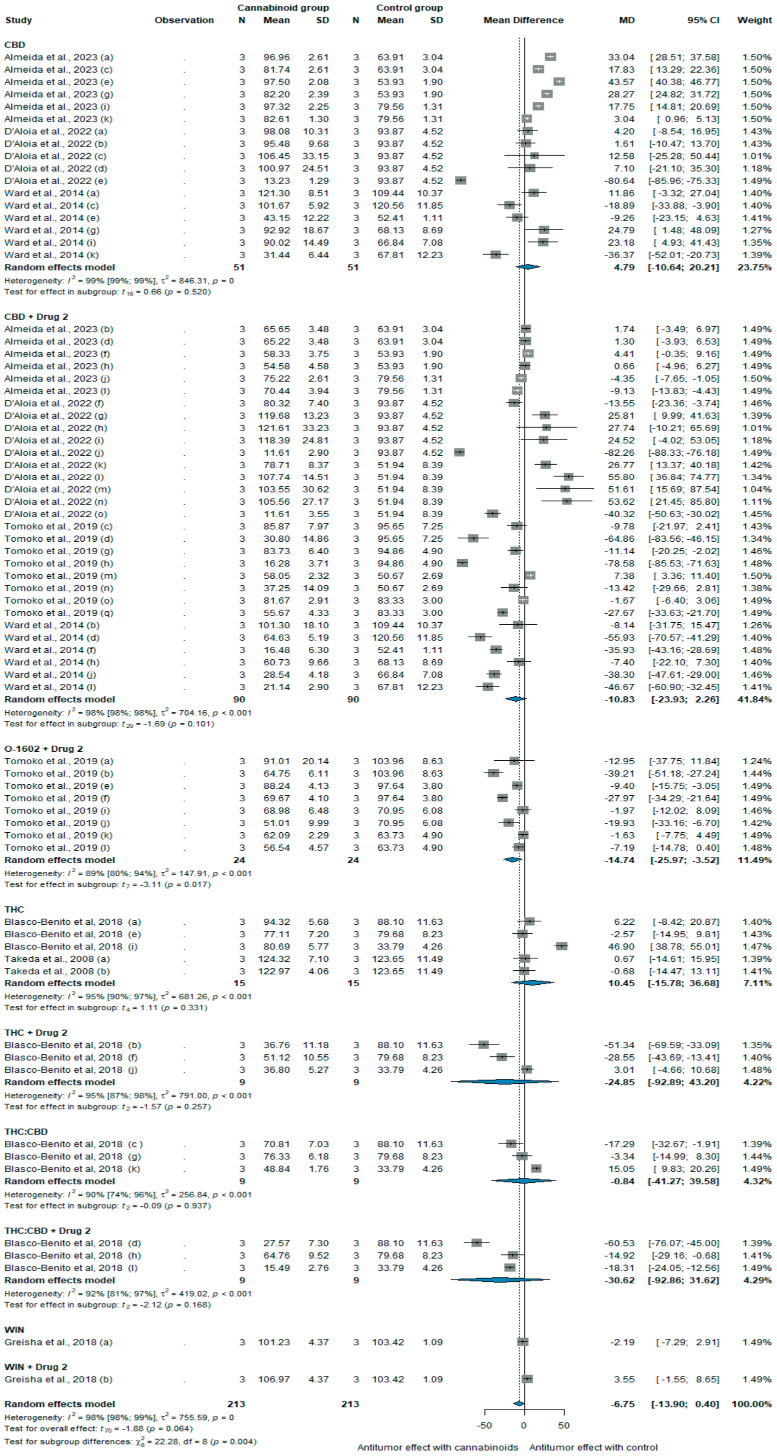

Figure S1. The effects of cannabinoids vs control in breast cellular models. Subgroup analyses are stratified by cannabinoid type (CBD, synthetic cannabinoids, THC, and THC:CBD combinations).

Abbreviations: CBD = cannabidiol; THC = Δ9-tetrahydrocannabinol; ; N=sample size; MD = mean difference; SD= standard deviation; CI = confidence interval.

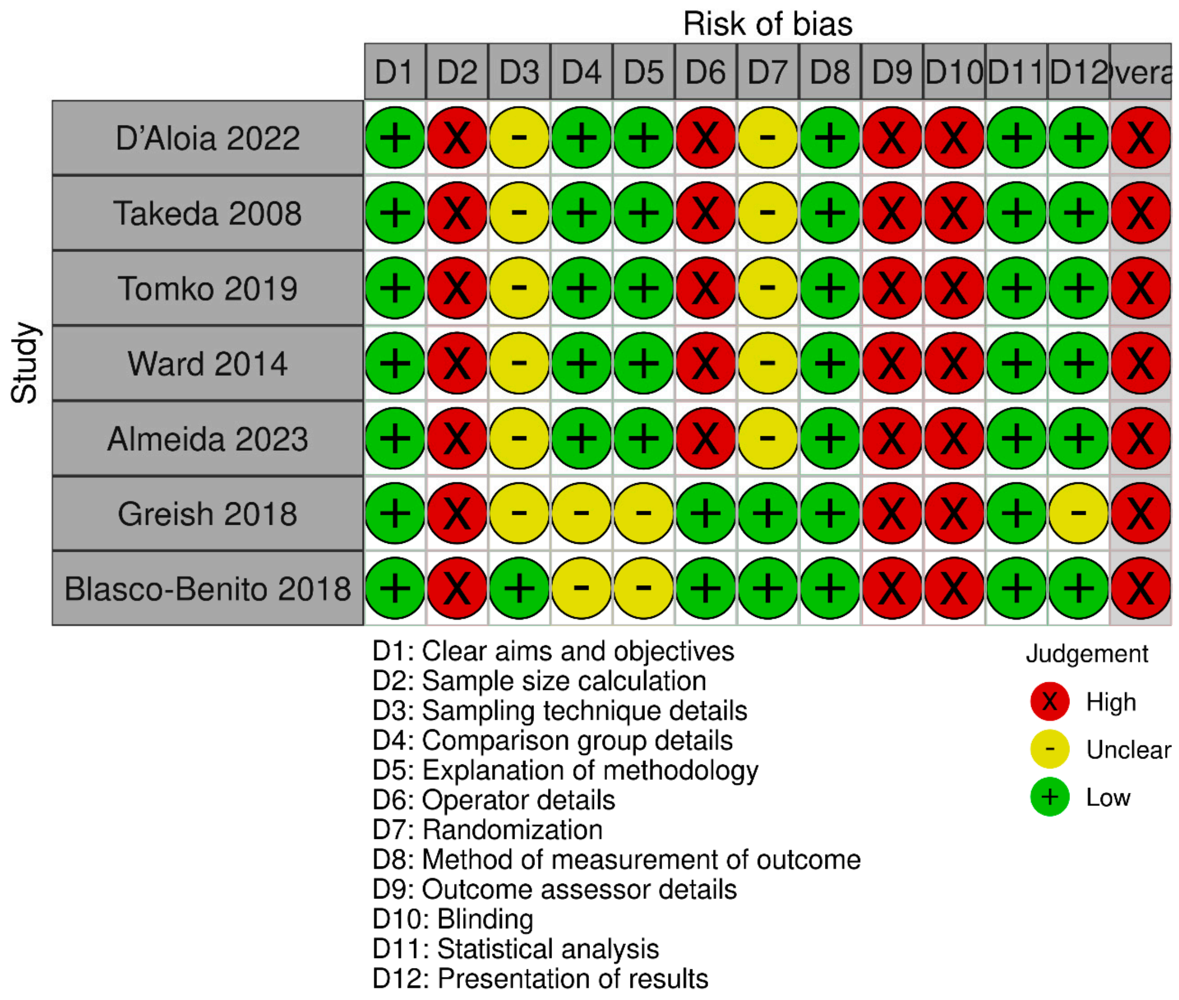

Figure S2. Risk of bias for in vitro breast cancer studies

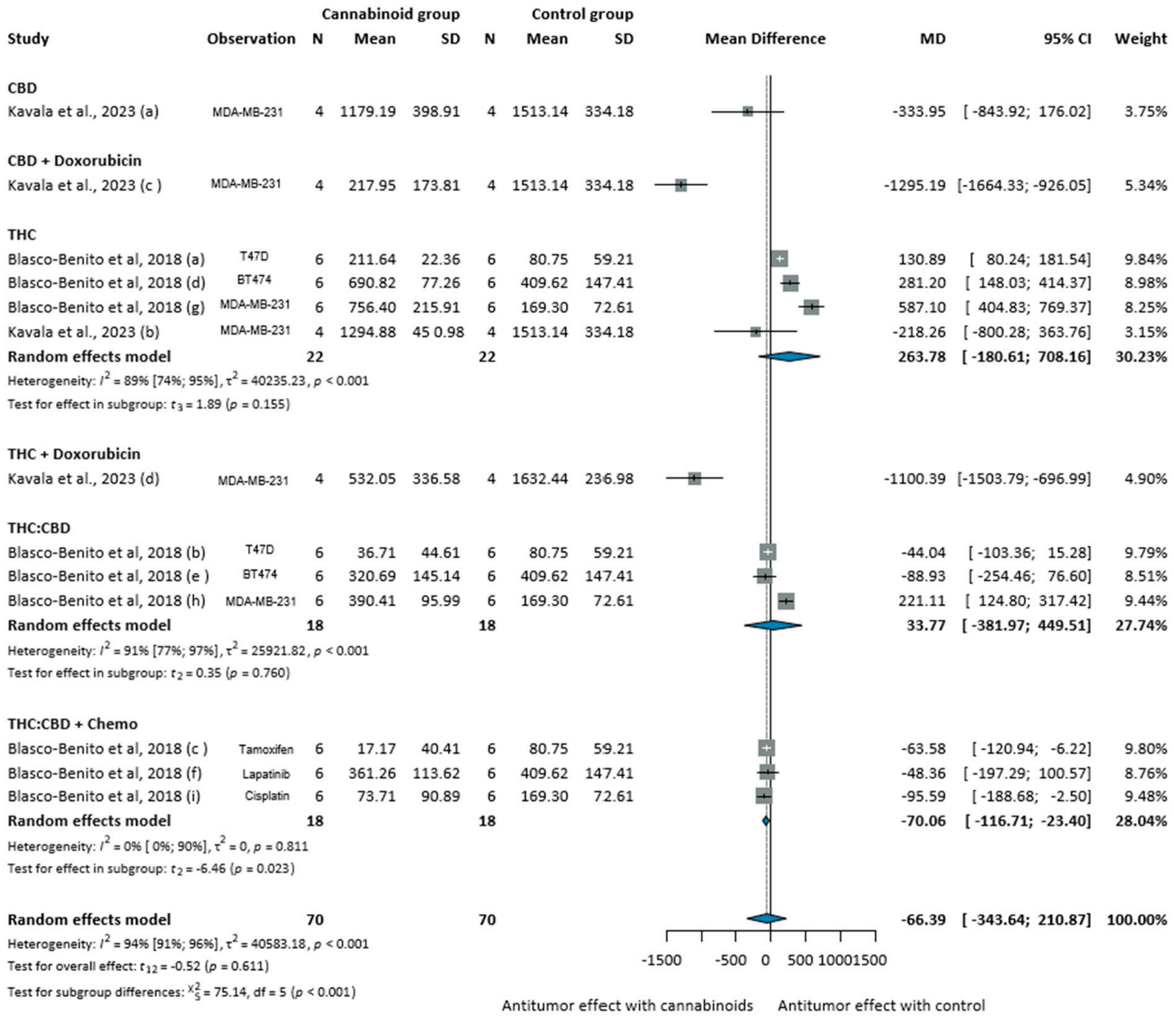

Figure S3. Effects of cannabinoids on breast cancer xenograft tumor volume (mm³). Forest plot illustrating the mean difference (MD) in tumor volume between cannabinoid alone or combined with chemotherapy and chemotherapy only treated animals. Subgroup analyses are stratified by cannabinoid type (CBD, synthetic cannabinoids, THC, and THC:CBD combinations). Abbreviations: CBD = cannabidiol; THC = Δ9-tetrahydrocannabinol; ; N=sample size; MD = mean difference; SD= standard deviation; CI = confidence interval.

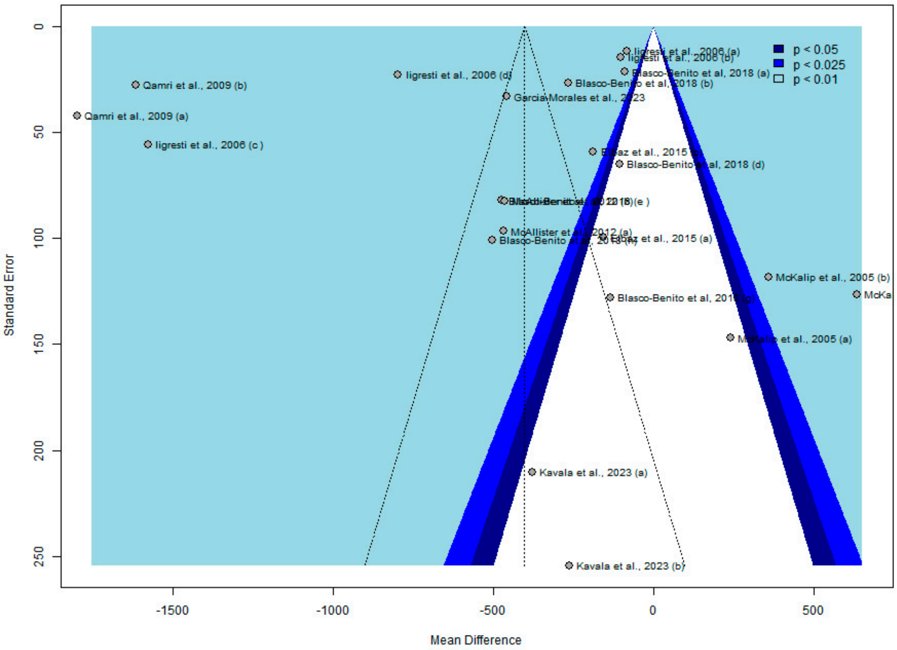

Figure S4. Funnel plot cannabinoid vs vehicle in breast cancer

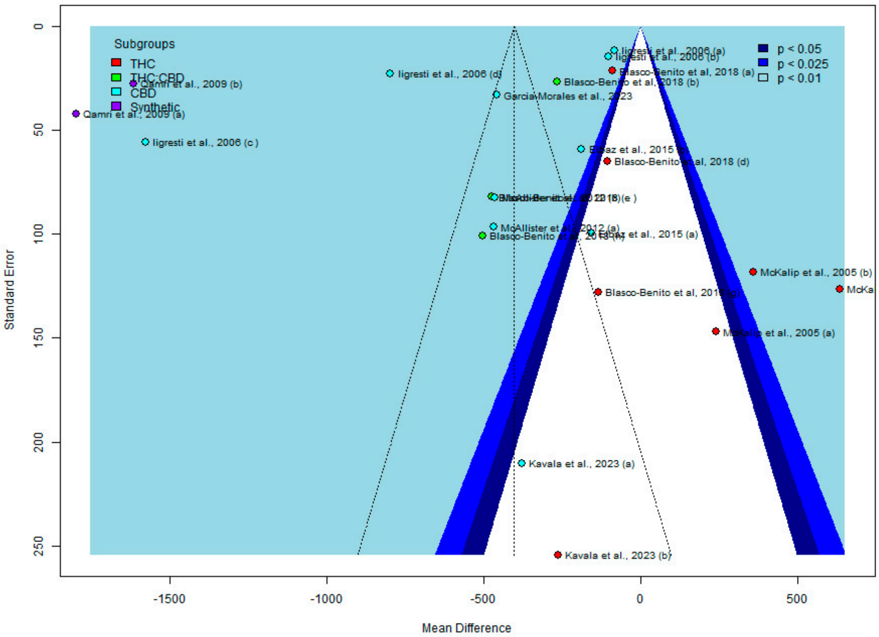

Figure S5. Funnel plot cannabinoid vs chemotherapy in breast cancer

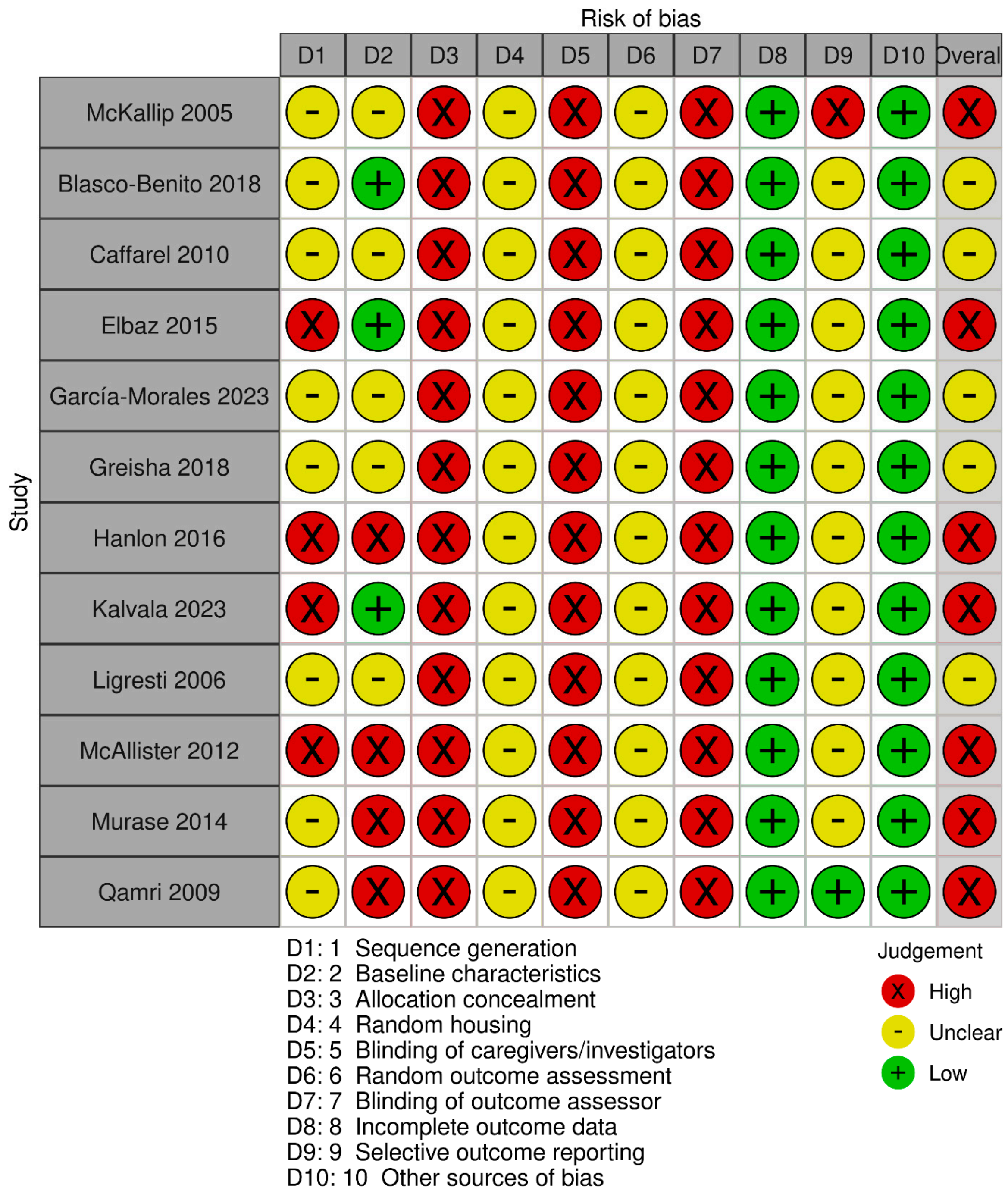

Figure S6. Risk of bias for in vivo breast cancer studies

2. Glioblastoma

Study	Observation	Cannabinoid group N	Cannabinoid group Mean	Cannabinoid group SD	Control group N	Control group Mean	Control group SD	MD	95% CI	Weight
**CBD**										
Huang et al, 2021 (a)	.	3	93.12	9.62	3	96.96	5.53	-3.84	[-16.40; 8.72]	3.21%
Huang et al, 2021 (b)	.	3	35.80	13.23	3	96.96	5.53	-61.16	[-77.39; -44.93]	3.03%
Soroceanu et al, 2022 (e)	.	3	101.72	19.04	3	95.91	10.06	5.81	[-18.55; 30.17]	2.59%
Soroceanu et al, 2022 (a)	.	3	106.43	12.35	3	90.91	5.07	15.52	[0.41; 30.63]	3.09%
Soroceanu et al, 2022 (c)	.	3	8.99	8.71	3	25.35	4.71	-16.36	[-27.57; -5.15]	3.26%
Soroceanu et al, 2022 (g)	.	3	48.85	6.82	3	52.58	3.98	-3.73	[-12.67; 5.21]	3.35%
Soroceanu et al, 2022 (i)	.	3	48.35	4.14	3	48.67	2.23	-0.32	[-5.64; 5.00]	3.45%
Soroceanu et al, 2022 (m)	.	3	54.67	5.21	3	105.76	6.98	-51.09	[-60.95; -41.23]	3.32%
Soroceanu et al, 2022(k)	.	3	62.83	4.05	3	63.56	2.89	-0.73	[-6.36; 4.90]	3.45%
**Random effects model**		**27**			**27**			**-13.01**	**[-32.77; 6.76]**	**28.74%**
Heterogeneity: $I^2 = 94\%$ [91%; 96%], $\tau^2 = 388.09$, $p < 0.001$										
Test for effect in subgroup: $t_8 = -1.52$ ($p = 0.168$)										
**CBD + Drug 2**										
Huang et al, 2021 (c)	.	3	87.90	8.42	3	96.96	5.53	-9.06	[-20.46; 2.34]	3.26%
Huang et al, 2021 (d)	.	3	12.63	0.89	3	96.96	5.53	-84.33	[-90.67; -77.99]	3.43%
Soroceanu et al, 2022 (b)	.	3	60.29	4.69	3	90.91	5.07	-30.62	[-38.44; -22.80]	3.39%
Soroceanu et al, 2022 (d)	.	3	10.03	4.38	3	25.35	4.71	-15.32	[-22.60; -8.04]	3.40%
Soroceanu et al, 2022 (f)	.	3	70.83	6.82	3	95.91	10.06	-25.08	[-38.84; -11.32]	3.15%
Soroceanu et al, 2022 (h)	.	3	7.21	4.76	3	52.58	3.98	-45.37	[-52.40; -38.34]	3.41%
Soroceanu et al, 2022 (j)	.	3	32.07	4.14	3	48.67	2.23	-16.60	[-21.92; -11.28]	3.45%
Soroceanu et al, 2022 (l)	.	3	60.27	6.10	3	63.56	2.89	-3.29	[-10.93; 4.35]	3.39%
Soroceanu et al, 2022 (n)	.	3	64.24	5.21	3	105.76	6.98	-41.52	[-51.38; -31.66]	3.32%
**Random effects model**		**27**			**27**			**-30.22**	**[-49.30; -11.13]**	**30.20%**
Heterogeneity: $I^2 = 98\%$ [97%; 99%], $\tau^2 = 764.15$, $p < 0.001$										
Test for effect in subgroup: $t_8 = -3.65$ ($p = 0.006$)										
**THC**										
Torres et al, 2011 (a)	.	6	98.36	4.36	6	99.91	4.63	-1.55	[-6.64; 3.54]	3.46%
Torres et al, 2011 (c)	.	6	98.36	4.36	6	92.94	2.17	5.42	[1.52; 9.32]	3.48%
Torres et al, 2011 (e)	.	6	89.98	1.39	6	102.43	7.35	-12.45	[-18.44; -6.46]	3.44%
Torres et al, 2011 (g)	.	6	89.98	1.39	6	90.29	3.25	-0.31	[-3.14; 2.52]	3.49%
Torres et al, 2011 (i)	.	6	94.95	10.06	6	92.43	2.46	2.52	[-5.77; 10.81]	3.37%
Torres et al, 2011 (k)	.	6	94.95	10.06	6	89.26	3.55	5.69	[-2.85; 14.23]	3.36%
**Random effects model**		**36**			**36**			**-0.26**	**[-7.21; 6.68]**	**20.60%**
Heterogeneity: $I^2 = 81\%$ [60%; 91%], $\tau^2 = 27.12$, $p < 0.001$										
Test for effect in subgroup: $t_5 = -0.10$ ($p = 0.926$)										
**THC + Drug 2**										
Torres et al, 2011 (b)	.	6	70.81	9.52	6	99.91	4.63	-29.10	[-37.57; -20.63]	3.37%
Torres et al, 2011 (d)	.	6	68.47	7.61	6	92.94	2.17	-24.47	[-30.80; -18.14]	3.43%
Torres et al, 2011 (f)	.	6	67.07	7.34	6	102.43	7.35	-35.36	[-43.67; -27.05]	3.37%
Torres et al, 2011 (h)	.	6	62.27	3.83	6	90.29	3.25	-28.02	[-32.04; -24.00]	3.48%
Torres et al, 2011 (j)	.	6	66.59	10.08	6	92.43	2.46	-25.84	[-34.14; -17.54]	3.37%
Torres et al, 2011 (l)	.	6	60.16	5.99	6	89.26	3.55	-29.10	[-34.67; -23.53]	3.45%
**Random effects model**		**36**			**36**			**-28.24**	**[-31.38; -25.10]**	**20.46%**
Heterogeneity: $I^2 = 0\%$ [0%; 75%], $\tau^2 = 0$, $p = 0.461$										
Test for effect in subgroup: $t_5 = -23.11$ ($p < 0.001$)										
**Random effects model**		**126**			**126**			**-18.77**	**[-27.15; -10.39]**	**100.00%**
Heterogeneity: $I^2 = 97\%$ [97%; 98%], $\tau^2 = 432.67$, $p < 0.001$										
Test for overall effect: $t_{29} = -4.58$ ($p < 0.001$)										
Test for subgroup differences: $\chi^2_3 = 91.21$, df = 3 ($p < 0.001$)										

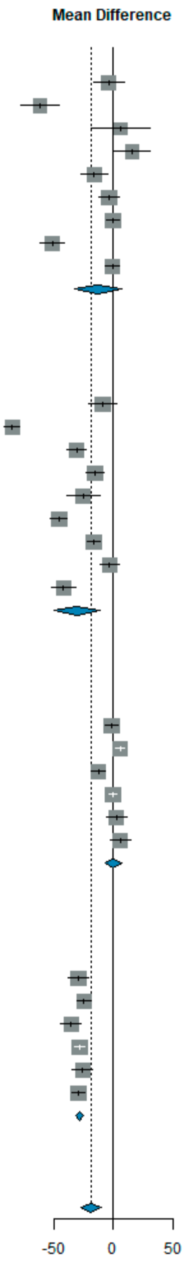

Antitumor effect with cannabinoids   Antitumor effect with control

Figure S7. The effects of cannabinoids vs control in glioblastoma cellular models. Abbreviations: CBD = cannabidiol; THC = Δ9-tetrahydrocannabinol; ; N=sample size; MD = mean difference; SD= standard deviation; CI = confidence interval.

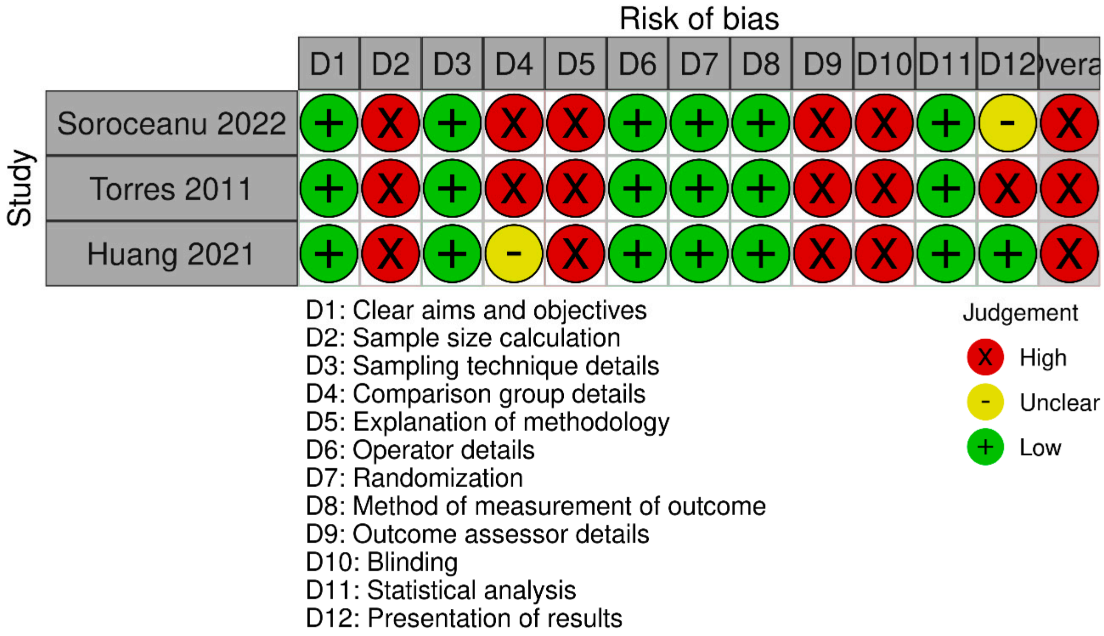

Figure S8. Risk of bias in vitro for glioblastoma studies

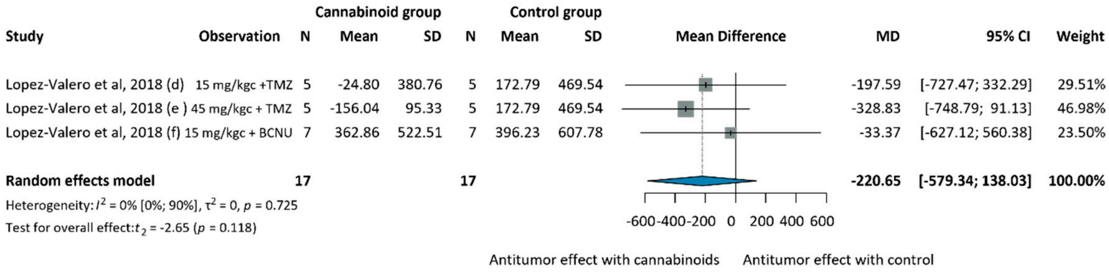

Figure S9. Effects of cannabinoids on glioblastoma xenograft tumor volume (mm³). Forest plot illustrating the mean difference (MD) in tumor volume between cannabinoid alone or combined with chemotherapy and chemotherapy only treated animals. Abbreviations: CBD = cannabidiol; THC = Δ9-tetrahydrocannabinol; ; N=sample size; MD = mean difference; SD= standard deviation; CI = confidence interval.

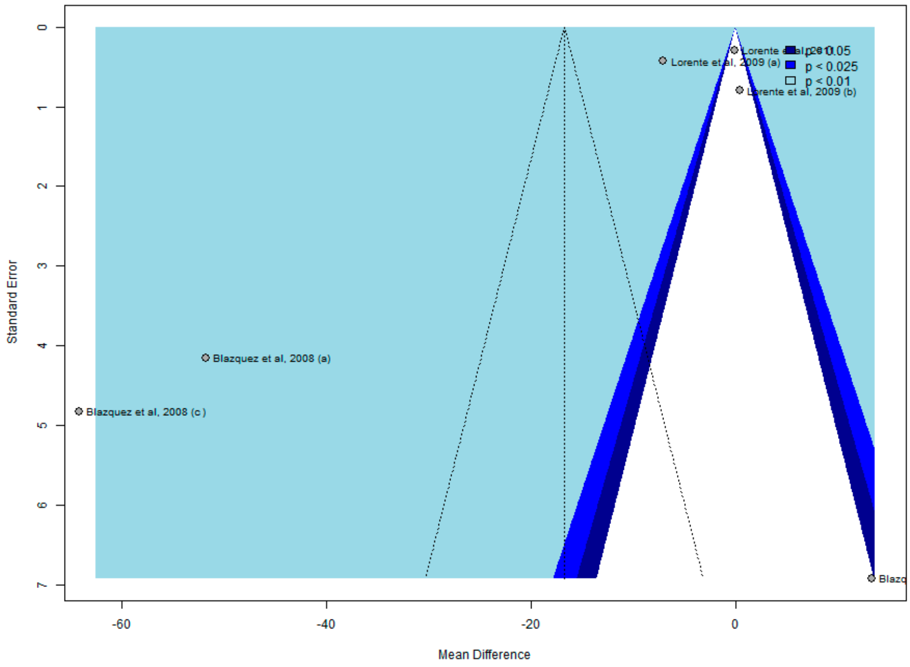

Figure S10. Funnel plot cannabinoid vs vehicle in glioblastoma

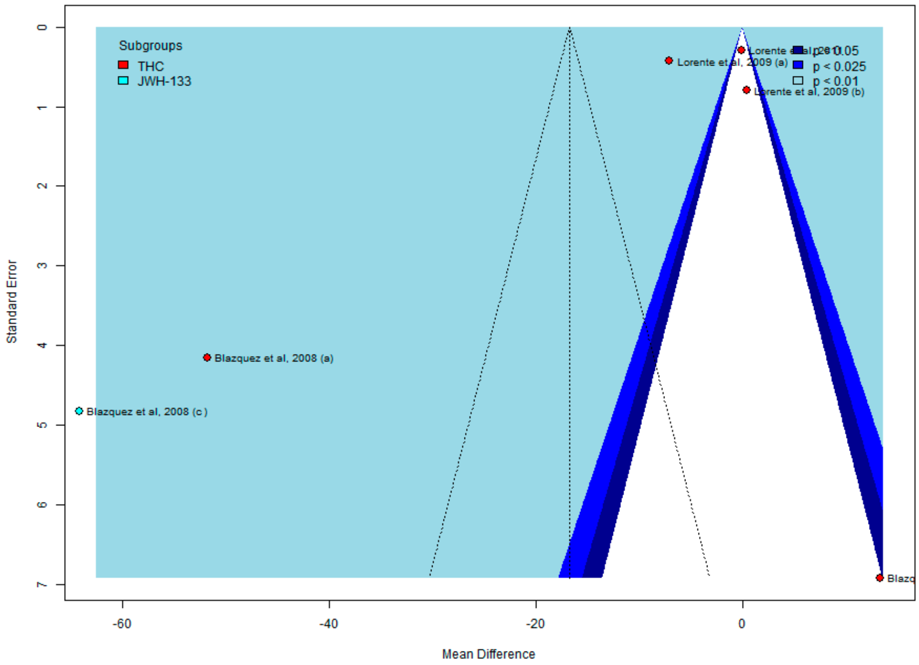

Figure S11. Funnel plot cannabinoid vs chemotherapy in glioblastoma

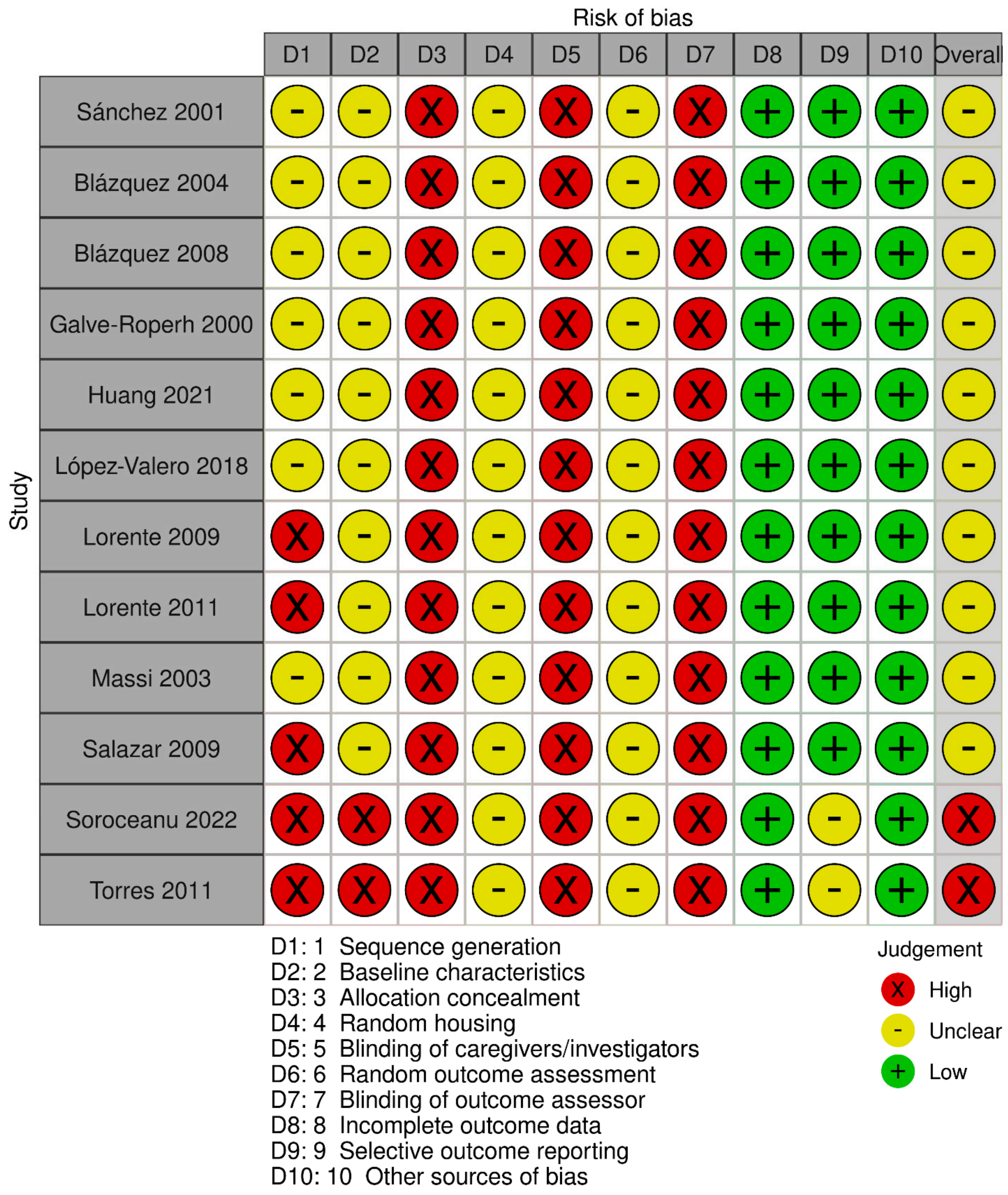

Figure S12. Risk of bias for glioblastoma studies

3. Lung cancer

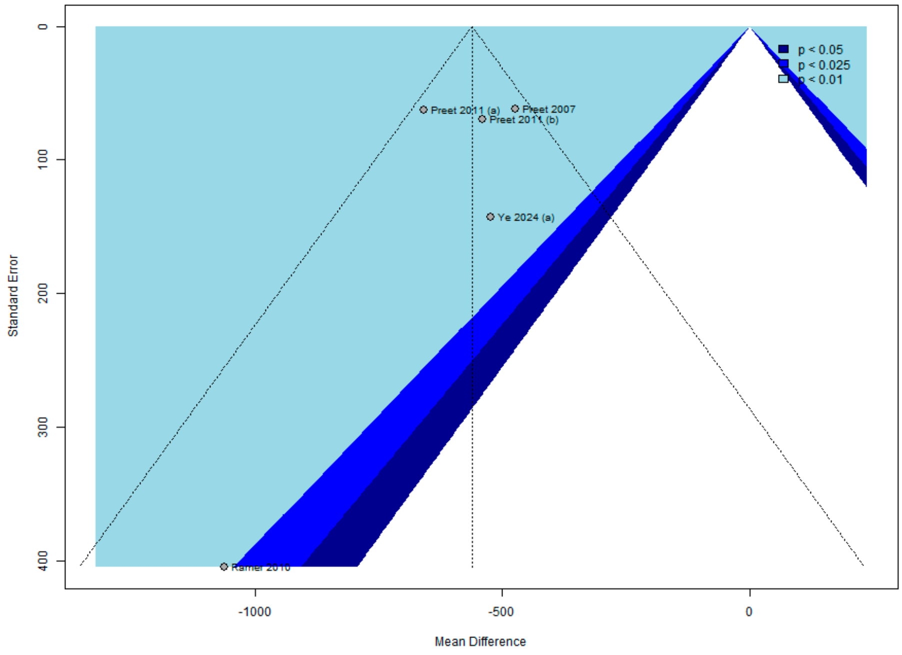

Figure S13. Funnel plot cannabinoid vs vehicle in lung cancer

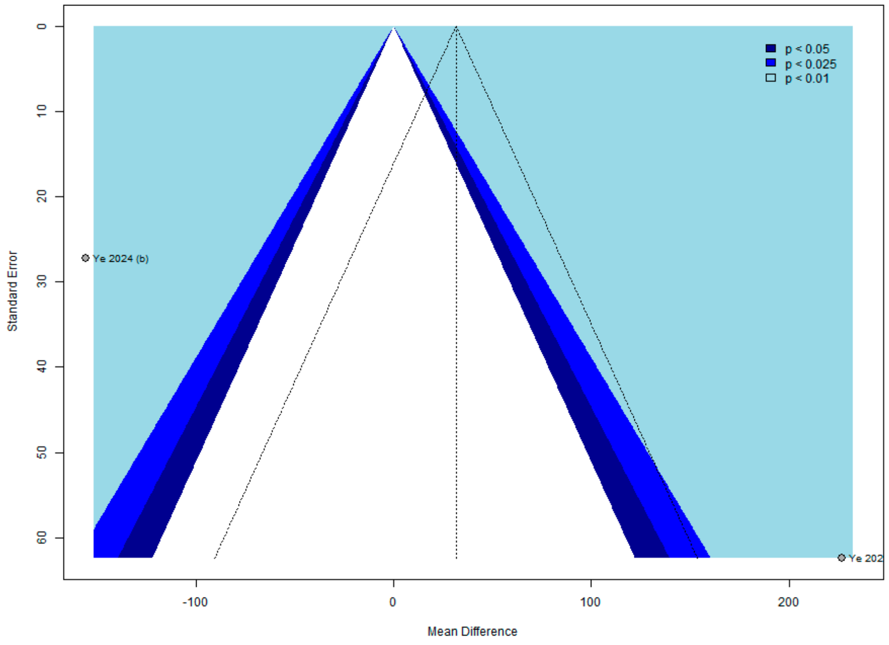

Figure S14. Funnel plot cannabinoid vs chemotherapy in lung cancer

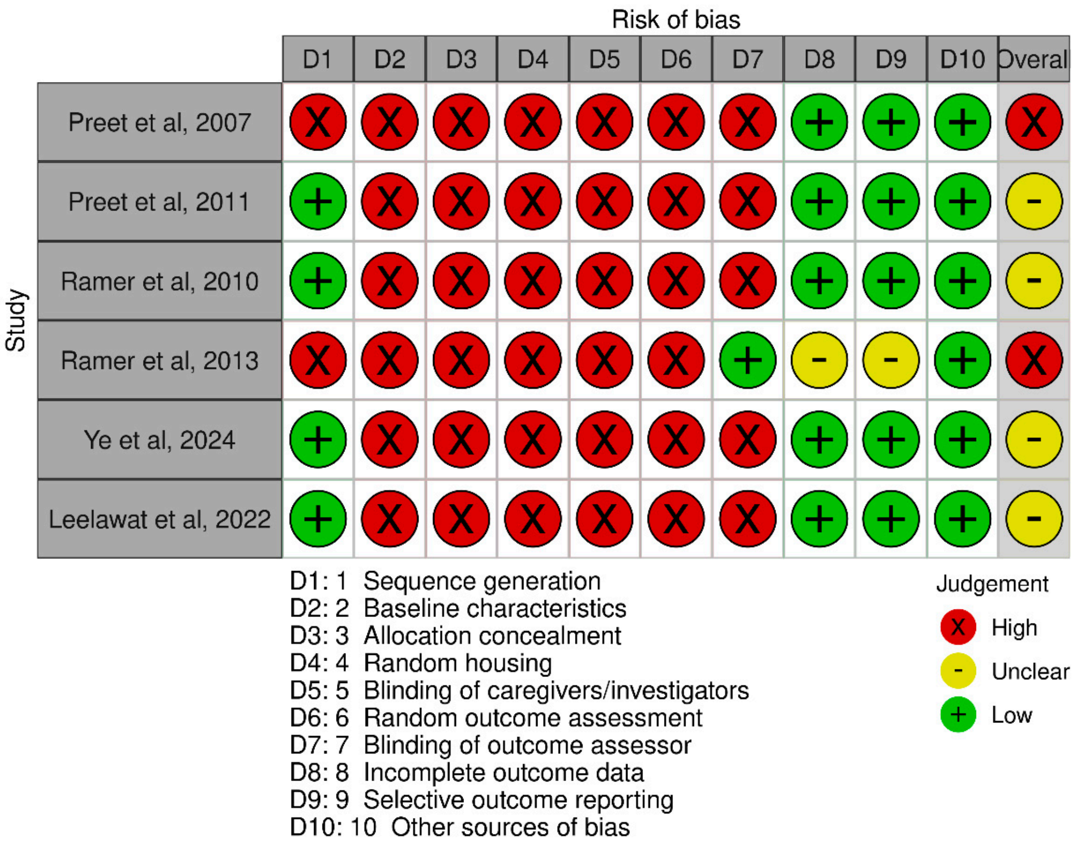

Figure S15. Risk of bias for lung cancer studies

4. Prostate cancer

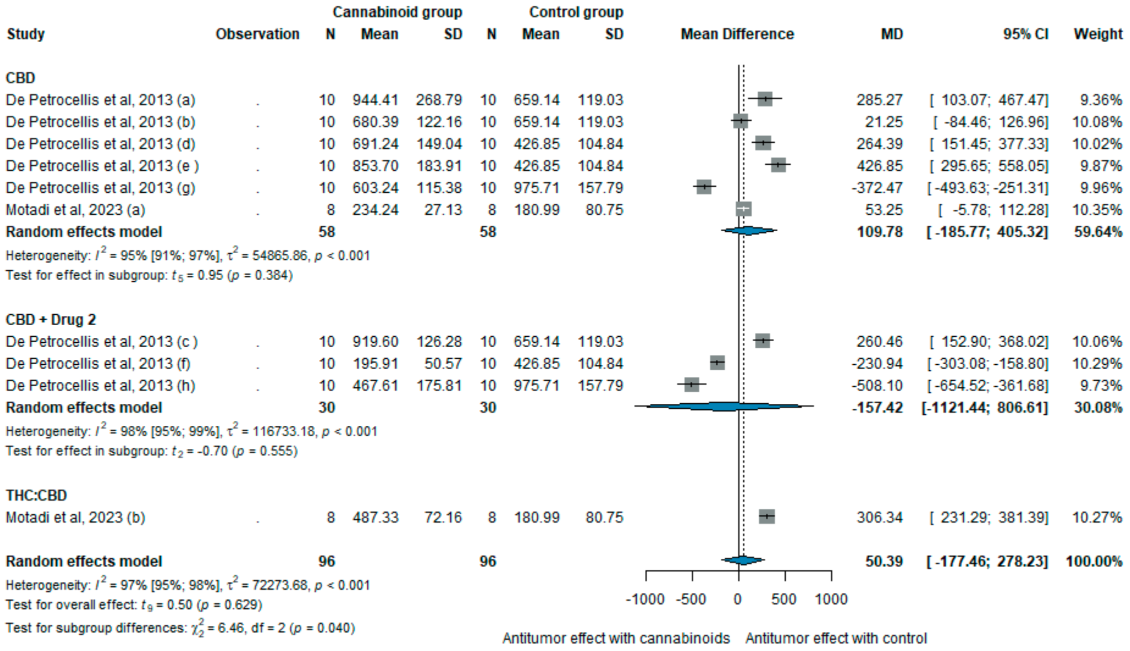

Figure S16. Effects of cannabinoids on prostate xenograft tumor volume (mm³). Forest plot illustrating the mean difference (MD) in tumor volume between cannabinoid alone or combined with chemotherapy and chemotherapy only treated animals. Abbreviations: CBD = cannabidiol; THC = Δ9-tetrahydrocannabinol; ; N=sample size; MD = mean difference; SD= standard deviation; CI = confidence interval.

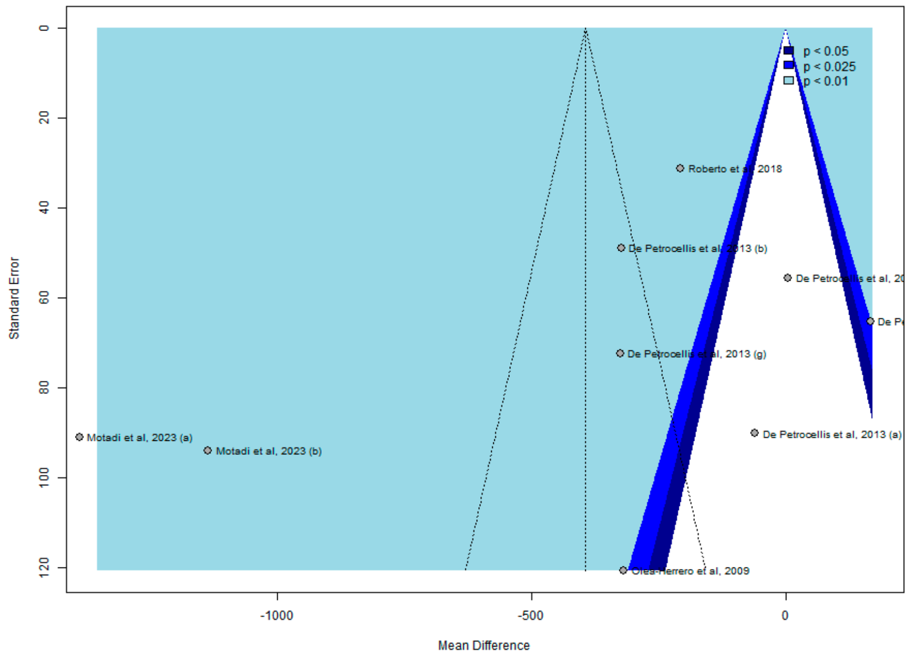

Figure S17. Funnel plot cannabinoid vs vehicle in prostate cancer

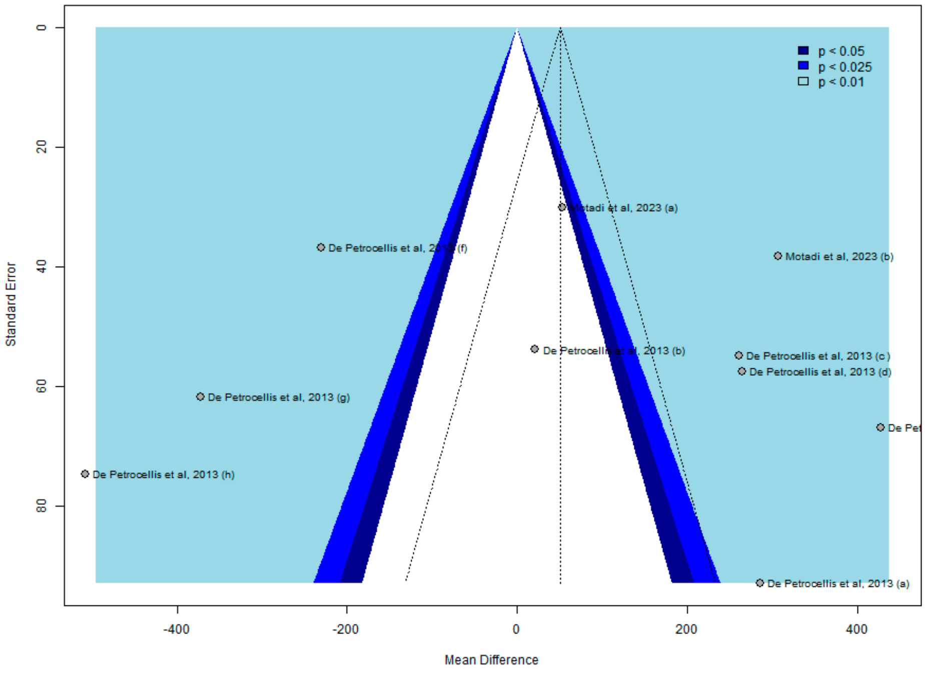

Figure S18. Funnel plot cannabinoid vs chemotherapy in prostate cancer

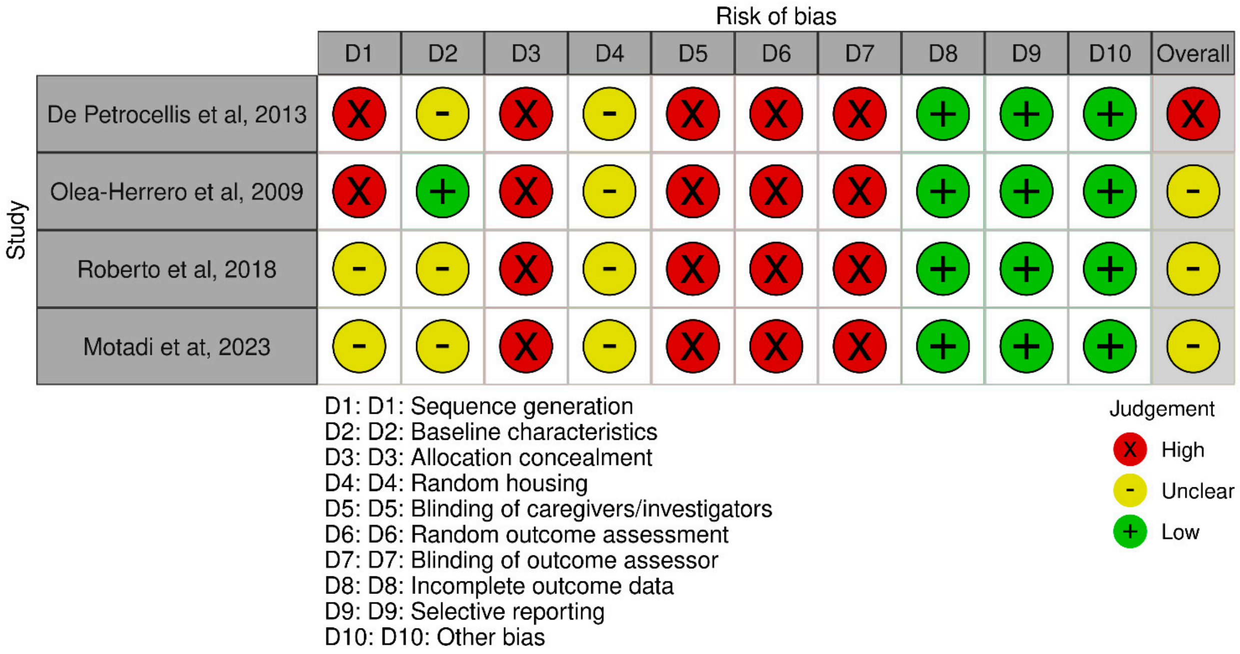

Figure 19. Risk of bias for prostate cancer studies

5. Colon cancer

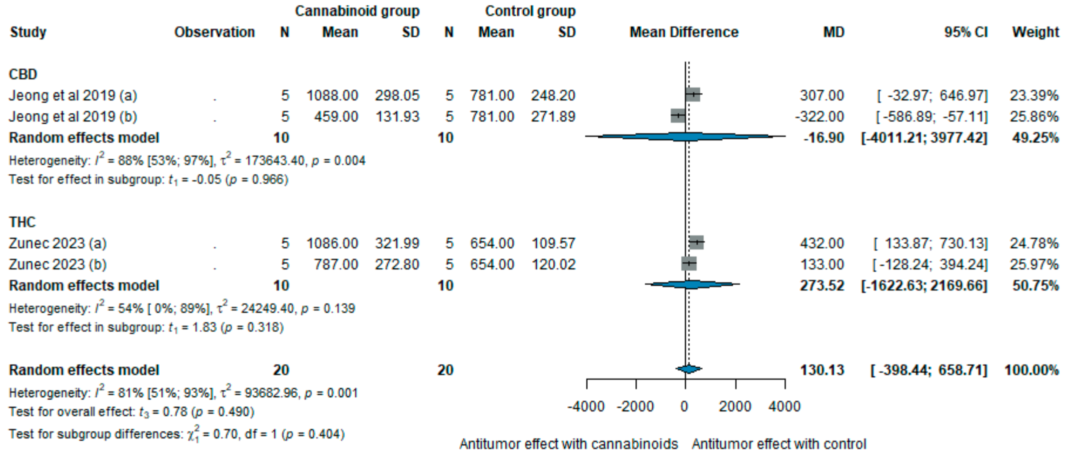

Figure S20. Effects of cannabinoids on colon cancer xenograft tumor volume (mm³). Forest plot illustrating the mean difference (MD) in tumor volume between cannabinoid alone or combined with chemotherapy and chemotherapy only treated animals. Abbreviations: CBD = cannabidiol; THC = Δ9-tetrahydrocannabinol; ; N=sample size; MD = mean difference; SD= standard deviation; CI = confidence interval.

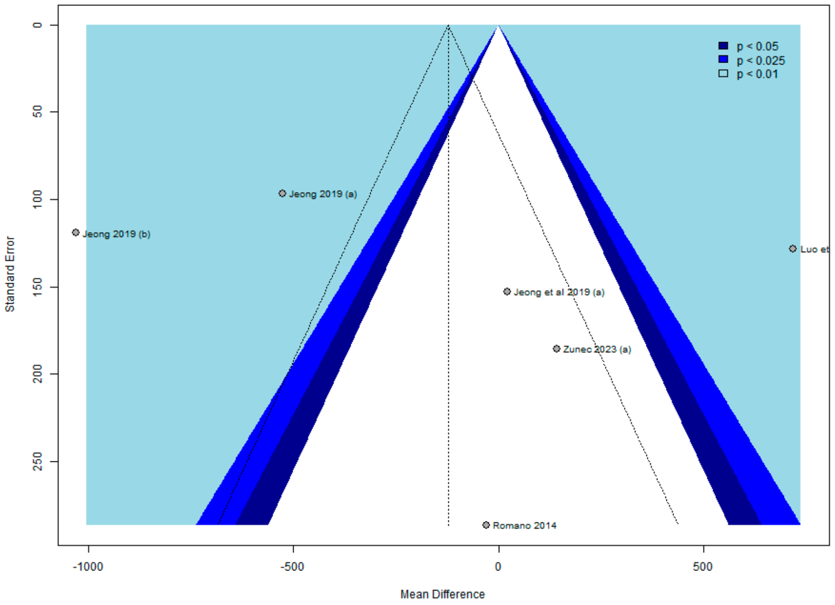

Figure S21. Funnel plot cannabinoid vs vehicle in colorectal cancer

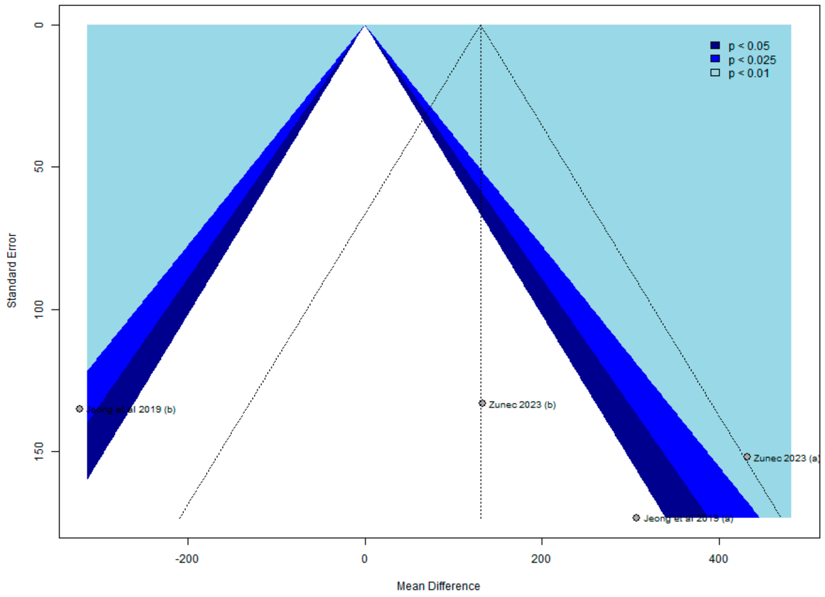

Figure S22. Funnel plot cannabinoid vs chemotherapy in colorectal cancer

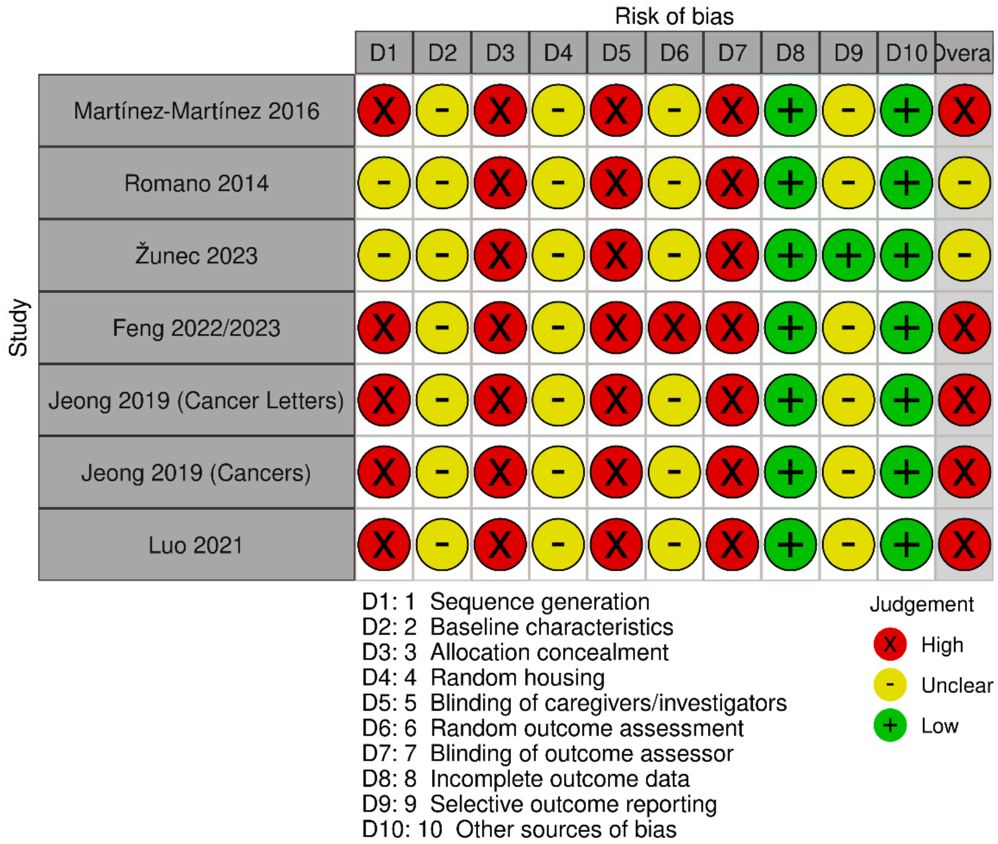

Figure S23. Risk of bias for colorectal cancer studies.

6. Hepatocarcinoma

Study	Observation	Cannabinoid group N	Mean	SD	Control group N	Mean	SD	MD	95% CI	Weight
**CBD**										
Shangguan et al, 2021	HepG2, 40 mg/kgc	4	467.71	206.03	4	929.66	143.68	-461.95	[-708.10; -215.80]	0.01%
**JWH-015**										
Vara et al, 2011 (b)	HepG2, 1.5 mg/kgc	8	4.43	2.81	8	11.81	2.75	-7.38	[-10.10; -4.66]	16.20%
Vara et al, 2011 (d)	Huh-7, 1.5 mg/kgc	8	4.21	1.77	8	12.83	2.47	-8.62	[-10.72; -6.52]	18.34%
Vara et al, 2013 (b)	HepG2, 1.5 mg/kgc	8	4.54	2.79	8	9.33	3.33	-4.79	[-7.80; -1.78]	15.23%
**Random effects model**		**24**			**24**			**-7.14**	**[-11.91; -2.36]**	**49.77%**
Heterogeneity: I^2 = 52% [0%; 86%] , ?^{2} = 1.90 , p = 0.124										
Test for effect in subgroup: t_2 = -6.43 (p = 0.023)										
**THC**										
Vara et al, 2011 (a)	Hep G2, 15 mg/kgc	8	3.90	2.21	8	11.81	2.75	-7.91	[-10.36; -5.46]	17.16%
Vara et al, 2011 (c)	Huh-7, 15 mg/kgc	8	6.97	2.47	8	12.83	2.47	-5.86	[-8.28; -3.44]	17.25%
Vara et al, 2013 (a)	Hep G2, 15mg/kgc	8	3.85	2.38	8	9.33	3.33	-5.48	[-8.32; -2.64]	15.81%
**Random effects model**		**24**			**24**			**-6.50**	**[-9.76; -3.24]**	**50.23%**
Heterogeneity: I^2 = 2% [0%; 90%] , τ^2 = 0.03 , p = 0.360										
Test for effect in subgroup: t_2 = -8.57 (p = 0.013)										
**Random effects model**		**52**			**52**			**-6.77**	**[-10.79; -2.76]**	**100.00%**
Heterogeneity: I^2 = 70% [34%; 86%] , τ^2 = 4.75 , p = 0.003										
Test for overall effect: t_6 = -4.13 (p = 0.006)										
Test for subgroup differences: χ^2_2 = 13.36, df = 2 (p = 0.001)										

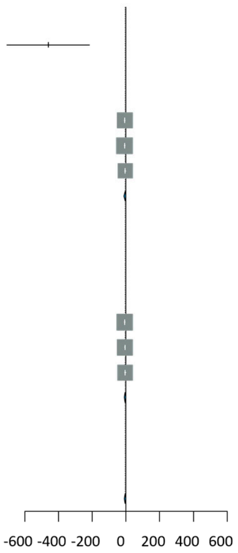

Figure S24. Effects of cannabinoids on hepatocarcinoma xenograft tumor volume (mm³). Forest plot illustrating the mean difference (MD) in tumor volume between cannabinoid-treated and vehicle-treated animals. Subgroup analyses are stratified by cannabinoid type (CBD, synthetic cannabinoids, THC, and THC:CBD combinations). Abbreviations: CBD = cannabidiol; THC = Δ9-tetrahydrocannabinol; ; N=sample size; MD = mean difference; SD= standard deviation; CI = confidence interval.

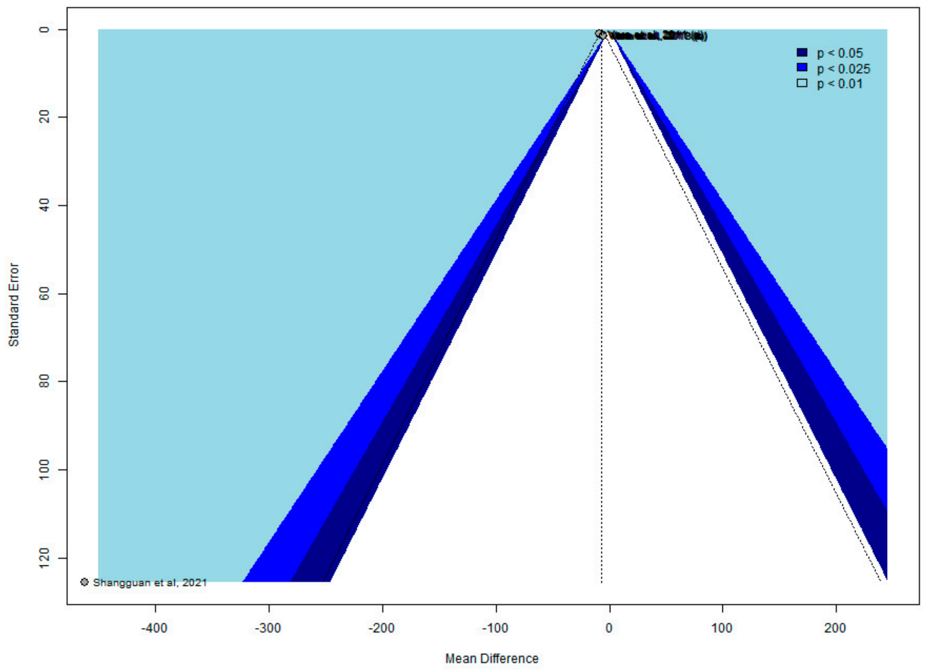

Figure S25. Funnel plot cannabinoid vs vehicle in hepatocarcinoma

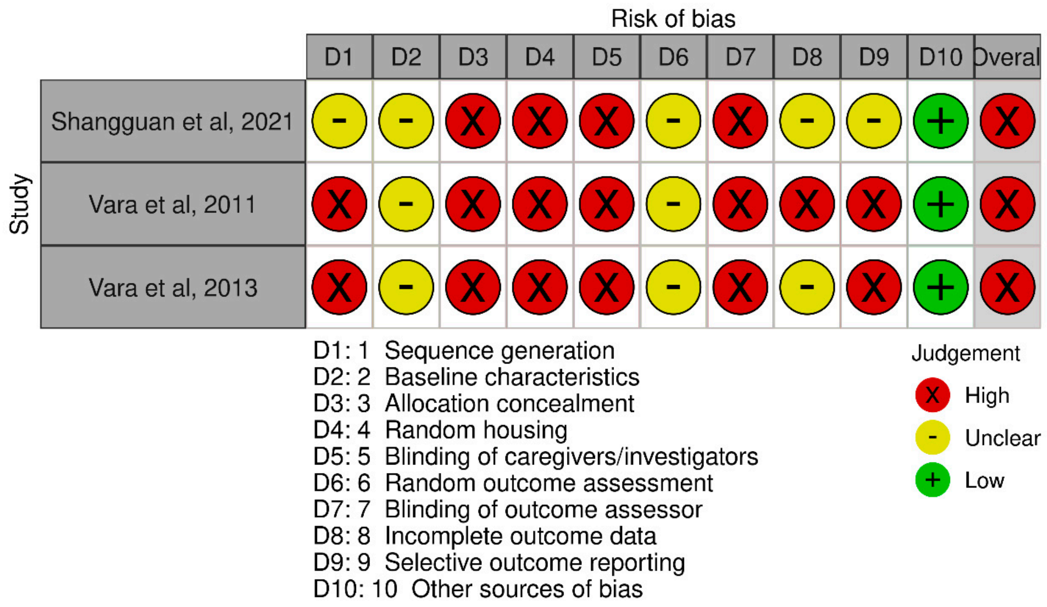

Figure S26. Risk of bias for colorectal cancer studies

7. Pancreatic cancer

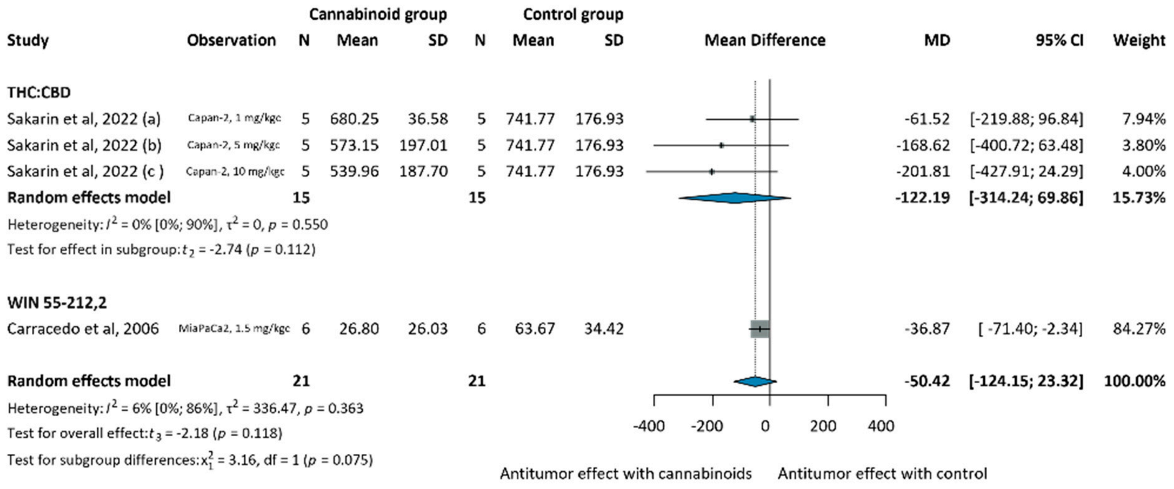

Figure S27. Effects of cannabinoids on pancreatic xenograft tumor volume (mm³). Forest plot illustrating the mean difference (MD) in tumor volume between cannabinoid-treated and vehicle-treated animals. Subgroup analyses are stratified by cannabinoid type (CBD, synthetic cannabinoids, THC, and THC:CBD combinations). Abbreviations: CBD = cannabidiol; THC = Δ9-tetrahydrocannabinol; ; N=sample size; MD = mean difference; SD= standard deviation; CI = confidence interval.

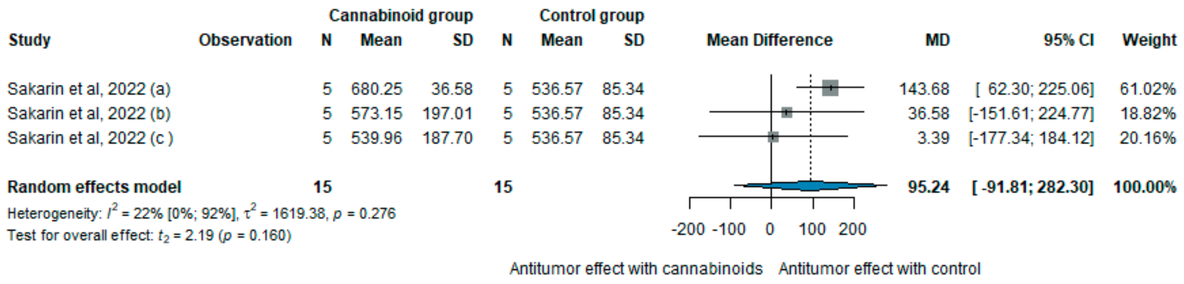

Figure S28. Effects of cannabinoids on pancreatic xenograft tumor volume (mm³). Forest plot illustrating the mean difference (MD) in tumor volume between cannabinoid alone or combined with chemotherapy and chemotherapy only treated animals. Abbreviations: CBD = cannabidiol; THC = Δ9-tetrahydrocannabinol; ; N=sample size; MD = mean difference; SD= standard deviation; CI = confidence interval.

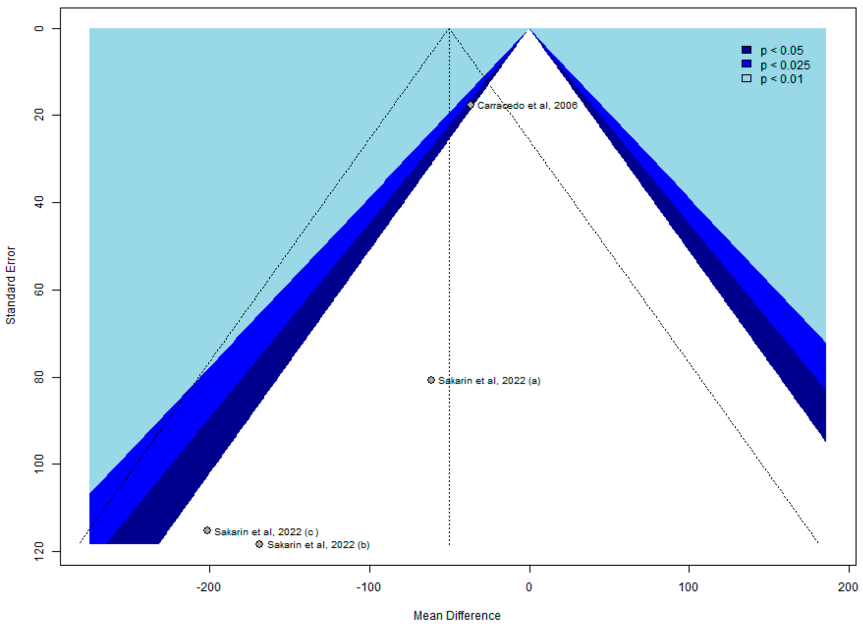

Figure S29. Funnel plot cannabinoid vs vehicle in pancreatic cancer

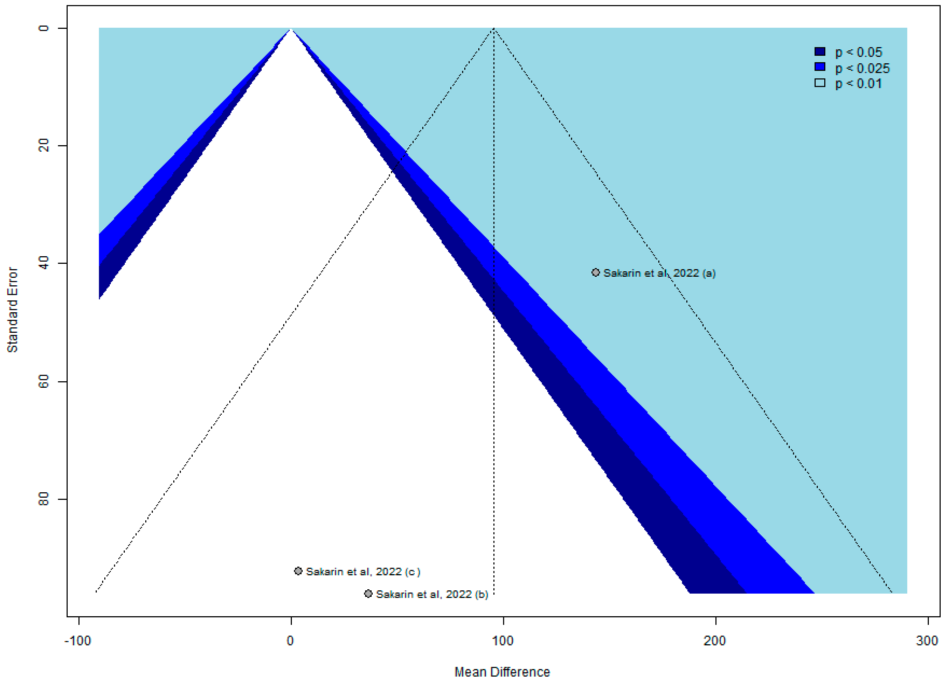

Figure S30. Funnel plot cannabinoid vs chemotherapy in pancreatic cancer

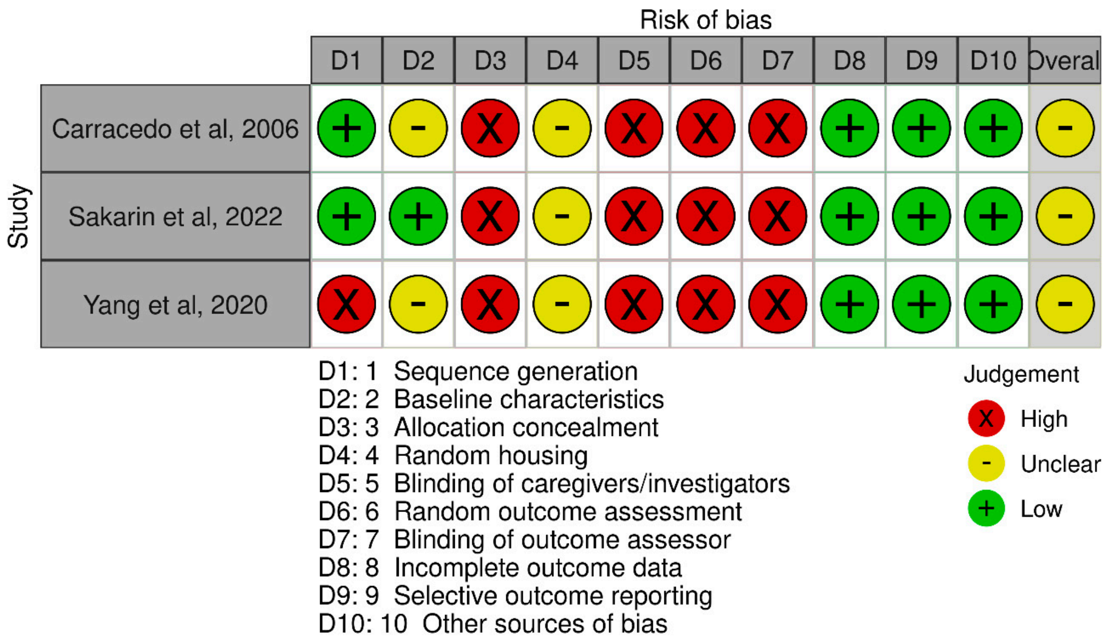

Figure S31. Risk of bias for pancreatic cancer studies

8. Skin cancer (including melanoma)

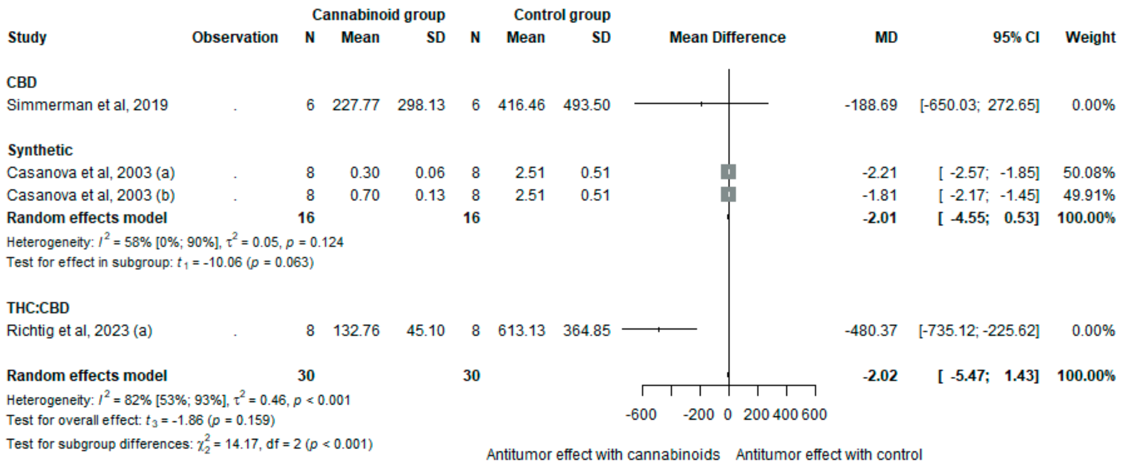

Figure S32. Effects of cannabinoids on skin xenograft tumor volume (mm³). Forest plot illustrating the mean difference (MD) in tumor volume between cannabinoid-treated and vehicle-treated animals. Subgroup analyses are stratified by cannabinoid type (CBD, synthetic cannabinoids, THC, and THC:CBD combinations). Abbreviations: CBD = cannabidiol; THC = Δ9-tetrahydrocannabinol; ; N=sample size; MD = mean difference; SD= standard deviation; CI = confidence interval.

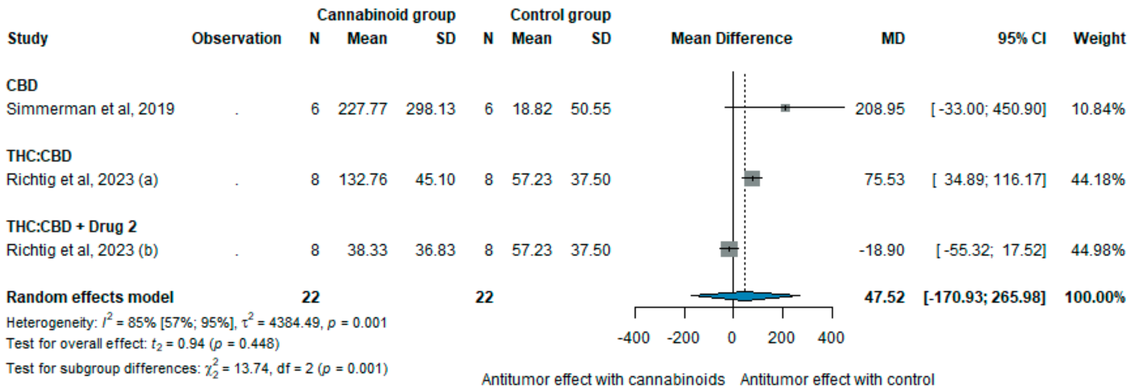

Figure S33. Effects of cannabinoids on skin xenograft tumor volume (mm³). Forest plot illustrating the mean difference (MD) in tumor volume between cannabinoid alone or combined with chemotherapy and chemotherapy only treated animals. Abbreviations: CBD = cannabidiol; THC = Δ9-tetrahydrocannabinol; ; N=sample size; MD = mean difference; SD= standard deviation; CI = confidence interval.

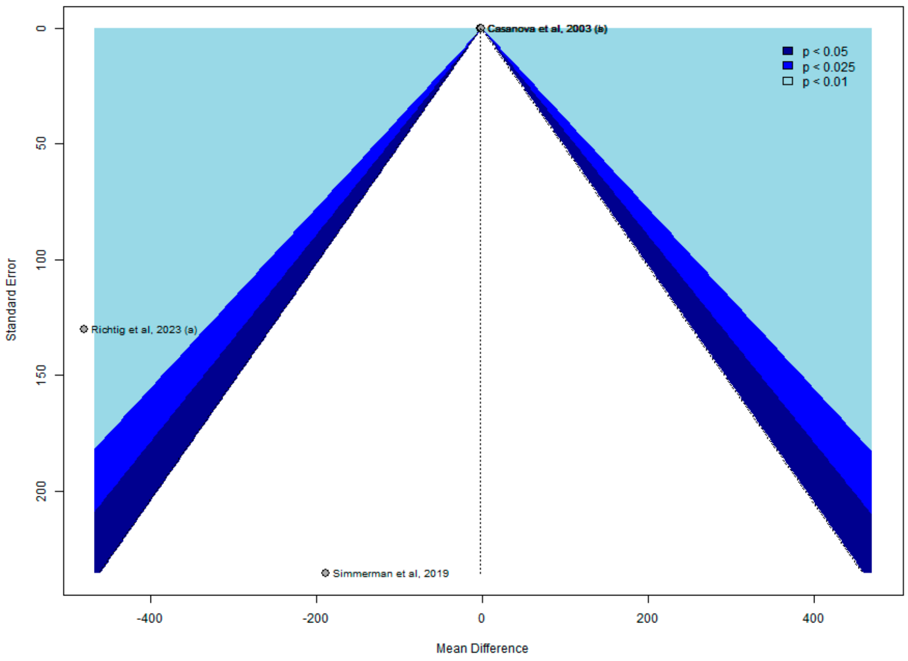

Figure S34. Funnel plot cannabinoid vs vehicle in skin cancer

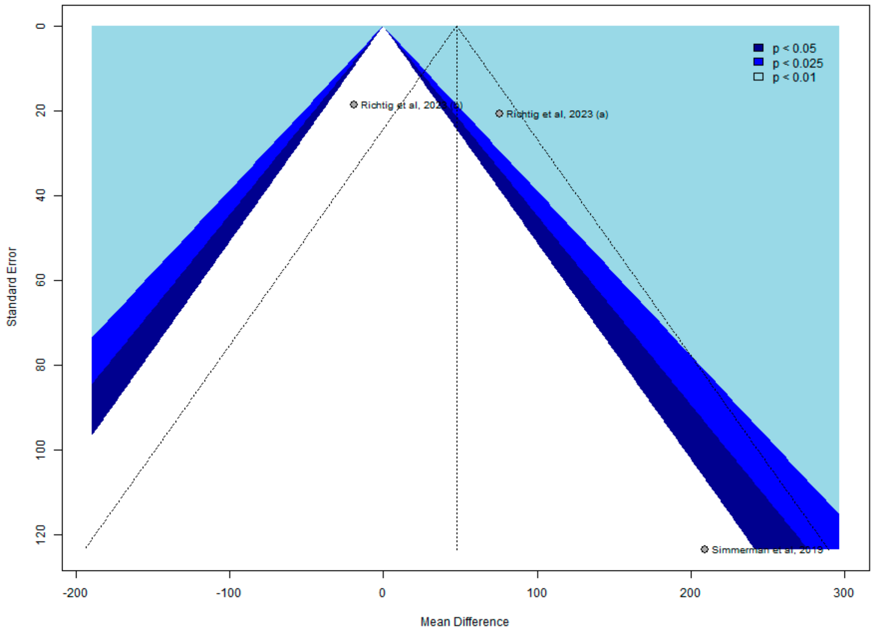

Figure S35. Funnel plot cannabinoid vs chemotherapy in skin cancer

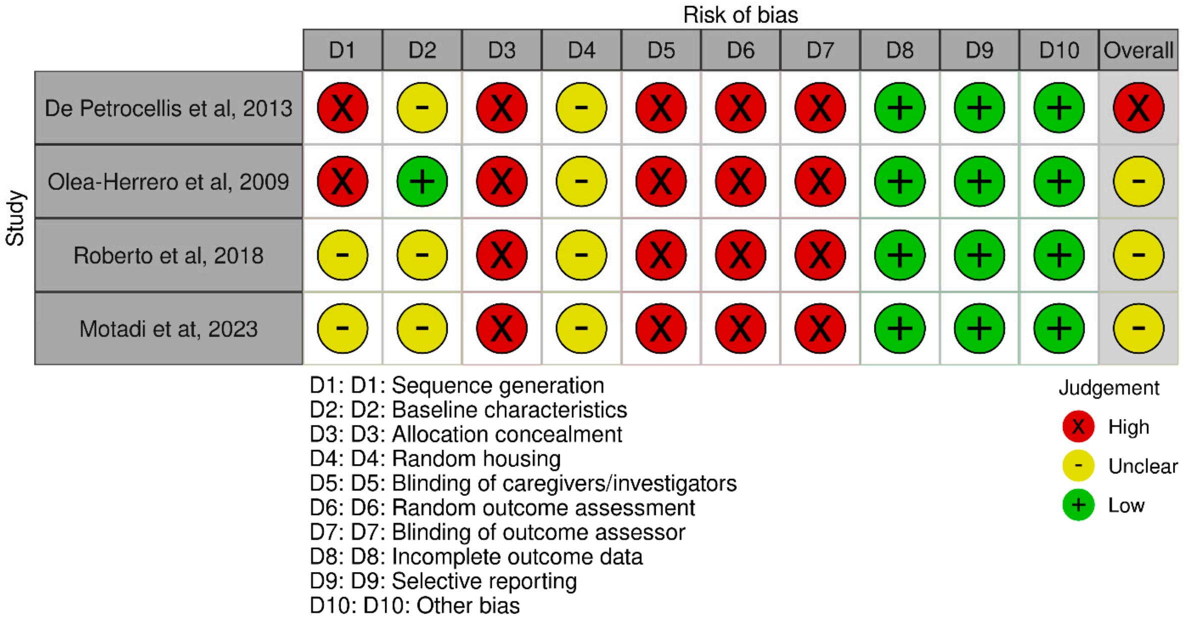

Figure S36. Risk of bias for skin cancer studies
